# Resilience of urban public electric vehicle charging infrastructure to flooding

**DOI:** 10.1038/s41467-022-30848-w

**Published:** 2022-06-09

**Authors:** Gururaghav Raman, Gurupraanesh Raman, Jimmy Chih-Hsien Peng

**Affiliations:** 1 Department of Electrical and Computer Engineering, National University of Singapore, 2 Engineering Drive 3, Singapore, 117581 Singapore; 2 Singapore-ETH Centre, Future Resilient Systems, CREATE campus, 1 CREATE Way, #06-01 CREATE Tower, Singapore, 138602 Singapore

**Keywords:** Sustainability, Climate-change impacts, Engineering

## Abstract

An adequate charging infrastructure is key to enabling high personal electric vehicle (EV) adoption rates. However, urban flooding—whose frequency and intensity are increasing due to climate change—may be an impediment. Here, we study how geographically-correlated outages due to floods impact public EV charging networks in Greater London. While we find no appreciable impact on the ability of battery EVs to serve typical urban driving behaviors, we observe disproportionate stresses on chargers both near, and surprisingly significantly farther from, the flooded regions. For instance, we find over 50% increase in charger utilization and 260% increase in the distance to the nearest available charger in parts of Greater London over 10 km away. Concerningly, the impact is most concentrated on already-stressed sections of the network, underscoring the infrastructure’s vulnerability. Finally, we develop and evaluate four strategies for city planners to enhance the flood resilience of cities’ public EV charging networks.

## Abstract (web summary)

A study of how the Greater London electric vehicle charging network is affected by flooding reveals disproportionate impacts on already-stressed parts of the network, peaking as far as over 10 km away from the flooded regions.

## Introduction

Battery electric vehicles (BEVs) continue to replace fossil-fuel driven ones in the global push to reduce carbon emissions in the transportation sector. A major associated challenge in realizing high BEV penetrations is ensuring an adequate support network of electric vehicle (EV) chargers; perceived and actual adequacy have been shown to significantly impact personal BEV adoption rates^1–6^. It is therefore essential to ensure that the charging infrastructure be resilient to external shocks. Here, we focus on one specific shock: urban flooding. Events in the recent past have shown that even developed cities can be vulnerable to different forms of flooding such as due to high tides and coastal flooding (e.g., Houston, 2017^7^), and river and surface water flooding (e.g., New York City^8^, London^9^, Singapore^10^, Zhengzhou^11^, all in 2021). Although floods usually disrupt predictable regions of a city, infrastructure upgrades may be hindered by financial constraints, or their benefits outpaced by the more frequent and ever-increasing impact from extreme weather events brought forth by climate change^12,13^.

In addition to affecting drivers’ ability to navigate roads during floods, which has been extensively studied previously^14–23^, flooding events make chargers in the affected zones unavailable to BEVs both within and outside. Floods can disrupt access to charging infrastructure in the following ways: (i) individual chargers may go out of service due to water damage; or, (ii) even if chargers were suitably weatherized or installed at a height as per siting recommendations (e.g., see ref. ^24^), they could be rendered unusable due to water logging at the parking area, presenting a hazard to potential users. In either case, the unique result of a flooding event is that it creates geographically correlated outages for a significant number of chargers in the network simultaneously, which under normal circumstances, is arguably unlikely. Here, we study how the charging patterns change during flooding events, and examine how the charging infrastructure could be made more resilient.

However, similar studies do not exist in the prior literature. On the one hand, the aforementioned studies^14–23^ on the impact of flooding on traffic do not touch upon BEVs and charging infrastructure. Similarly, those on multi-infrastructure cascading effects (e.g., refs. ^25–29^) analyze the simultaneous impact of floods on urban transportation, power, energy, and water networks, but have not yet considered the rapidly expanding EV charging infrastructure. On the other hand, some studies (e.g. ref. ^1,30^) examine the adequacy of charging infrastructure given a particular BEV penetration level but do not study the impact of flooding. Finally, while some research^31,32^ and policy papers^24,33^ advocate not siting chargers in areas that are prone to flooding, this may not always be feasible, particularly in key commercial and population centers, and as flood zones expand over time due to climate change. Overall, the prior works offer no insights as to the disruptions that may occur if significant fractions of the existing chargers are flooded. Repeated and geographically correlated disruptions could exacerbate user concerns regarding charger adequacy and discourage BEV adoption, given that convenience of charging^4,5^ and availability of public chargers^6^ have been shown to be some of the most significant factors in the initial adoption and continued use of BEVs.

When studying the impact of flooding, we are mainly concerned with public EV chargers due to the following reasons. First, public chargers, particularly those on-street, are more likely than private chargers (e.g., within residences, where homeowners are more likely to flood-proof their own premises) to flood. Second, while private chargers typically serve individual BEVs, public ones are generally optimally sited based on considerations such as traffic patterns^34–38^, power system loading constraints^34,35,39^, and proximity to attractions where drivers can spend the charging time^40^. They are essential for drivers without residential chargers^41^, and rapid chargers in particular have been shown to alleviate range anxiety^2,42^. This means that if a subset of the public chargers were to become unavailable, then the impact would likely be felt by a broader set of BEV users.

In this paper, we examine the impact of flooding on the present-day public charging infrastructure in Greater London. We chose this case study for three reasons: (i) the region presently accounts for about a seventh of all EV sales (BEVs and hybrid EVs) in the United Kingdom and plans to have only BEV sales by 2030, when 30% of the total vehicle stock is expected to be electric^30^; (ii) significant parts of the Greater London area remain vulnerable to flooding^13,43^. Specifically, 9 out of the 32 boroughs as well as the City of London have at least 10% (and up to 18%) of their land areas at “high risk” (>3.3% risk per year) according to the Greater London Authority, considering various types of flooding and existing flood defenses^13^. A further 8 out of the 50 Opportunity Areas—areas identified for large-scale future development—have over 10% (and up to 17%) of their land areas at high risk^13^; and, (iii) over 40% of drivers rely on public/on-street parking^30^, which are at the highest risk from flooding events. The key finding in our study is that coordinated large-scale disruptions brought about by urban flooding do not impact the adequacy of BEVs to serve typical urban driving behaviors, but rather, place disproportionate stresses and decrease accessibility in parts of the charging network. The impact, we find, is not only felt in the near-vicinity of the flooded region, but as far as 10–13 km away, propagating as BEV users defer their charging to later trips. Based on our results, we put forth four possible mitigation strategies, and report on their success in reducing the stress on the chargers, improving access, and in increasing the success of personal vehicle electrification.

## Results

### Disproportionate impact of flooding on EV charging network

**Fig. 1:**
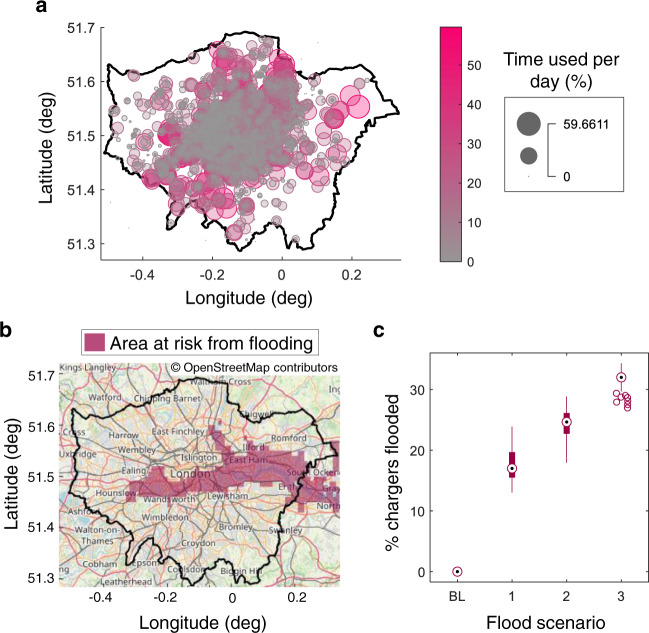
Vulnerability of electric vehicle chargers in Greater London to flooding. **a** Mean utilization levels of the public chargers in Greater London, considering 6 battery electric vehicles per charger and no flooding. **b** Regions at risk from flooding. **c** Fraction of chargers affected by flooding in the three flooding scenarios. The baseline scenario, marked as `BL’, corresponds to the case with no flooding. Results in **a** and **c** are shown as an average over 100 simulations where the driving patterns and flooded chargers were varied randomly. In **c**, the central mark, bottom, and top edges of each box plot represent the median, 25th, and 75th percentiles, respectively. Outliers, if any, are marked as individual circles.

We simulated personal BEV trips in Greater London using publicly available data on vehicle driving patterns, and assessed the usage of the 5925 public chargers presently operating in the region (see Methods). Our results are plotted in Fig. 1a, showing the utilization level of each charger, i.e., the fraction of the day when a BEV is connected to it. We then assess how flooding events impact these charging patterns. To this end, we divide the Greater London region into uniform grids and identify those grids that are at risk from flooding using data from Climate Central (see Methods); these are highlighted in Fig. 1b. We consider three different scenarios, referred to as scenarios-1, 2, and 3, that reflect progressively increasing intensities of flooding. Corresponding to these scenarios, an at-risk grid has a probability 0.5, 0.7, or 0.9 of being flooded, respectively. Subsequently, if a grid floods, the public chargers located within it are taken out of service, see Fig. 1c. We find that increasing the intensity of flooding only has a small impact on the number of BEV rides that are successful, i.e., those that are able to complete their planned journeys while maintaining a minimum state-of-charge (SOC) throughout, see Methods and Supplementary Note 1. In all cases, more than 99.7% of the rides are successful, an observation perhaps explained by our focus on only intra-city travel. Nevertheless, while such high success rates have indeed been reported by previous studies under normal circumstances (e.g., see ref. ^1^), it is notable that this remains the case despite over 34% of the public chargers being taken off service due to flooding.

**Fig. 2:**
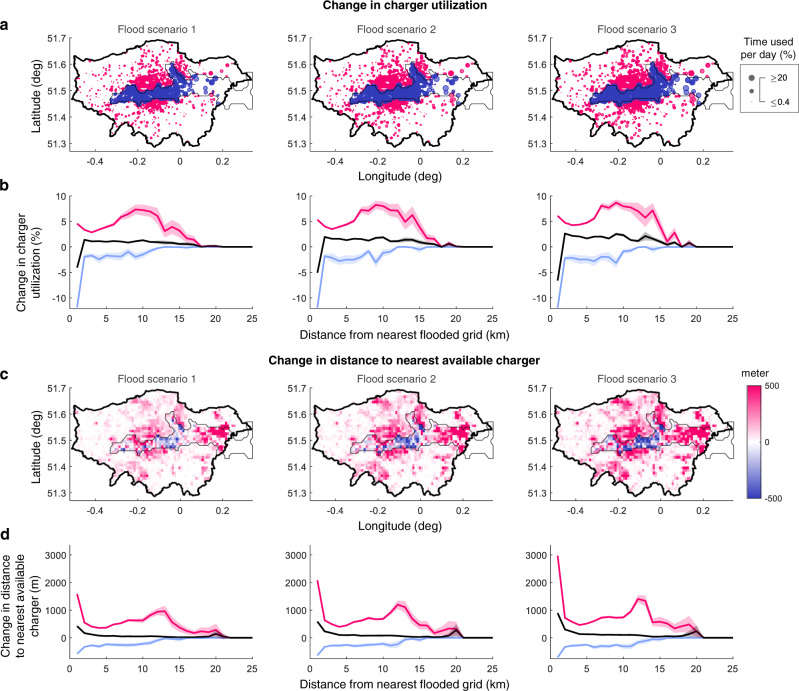
How flooding impacts battery electric vehicle (BEV)-charging behaviors. **a** Change in the chargers’ utilization across Greater London due to flooding. Chargers where utilization increased and decreased, are marked in red and blue, respectively. Regions at risk from flooding shown in Fig. 1b are encircled in black. **b** How the mean change in charger utilization in each grid varies with the distance from the nearest flooded grid. **c** Change in the distance from BEV users’ intended destinations to the nearest available chargers due to flooding, as a sum within each grid. **d** Same as **b**, but for the distance to the nearest available charger. All results are shown as an average over 100 simulations where the driving patterns and flooded chargers were varied randomly. In **b** and **d**, the black plot considers all locations, whereas the red and blue plots correspond to only those locations where the corresponding metric increased and decreased, respectively. The shaded areas in each plot represent the 95% confidence intervals.

We now assess the impact of flooding on the charging infrastructure. For this, we use two metrics: (i) the charger utilization level, which is the percentage of the day when a charger is used by a BEV; and (ii) the distance between the intended destination of a BEV user and the nearest available charger. While the first measures the stress on the charging infrastructure, the second captures the BEV users’ ability to access chargers near their destinations, and in turn, reflects their comfort level. Broadly, we find that the mean charger utilization across the entire network reduces during a flooding event (see Table 1) reflecting the fact that a section of the chargers transitions offline. Meanwhile, the maximum charger utilization increases, implying that while certain chargers experience a reduced usage, others experience increased stress. To examine this in more detail, we plot the change in the two metrics in Fig. 2a, c for the three flood scenarios, considering the baseline as the scenario with no flooding. To also observe how the disruption propagates geographically, the same values have been plotted in Fig. 2b, d, respectively, as a function of the distance from the nearest flooded grid. We note here that the charger utilization values are presented as an average over the different chargers in a grid, and the distance to the nearest available charger is shown as a sum over all BEV destinations in the grid. Furthermore, the overall trend (shown in black) for each metric is disaggregated into grids that experience a reduction (blue) and those that experience an increase in the metric (red).

**Table 1:** Impact of flooding on charger utilization levels.

	Maximum charger utilization (%)	Minimum charger utilization (%)	Mean charger utilization (%)	Standard deviation (%)
Baseline (no flooding)	59.7	0	7.9	10.1
Flood scenario-1	62.7	0	7.7	10.0
Flood scenario-2	63.2	0	7.5	10.3
Flood scenario-3	64.9	0	7.2	10.8

**Fig. 3:**
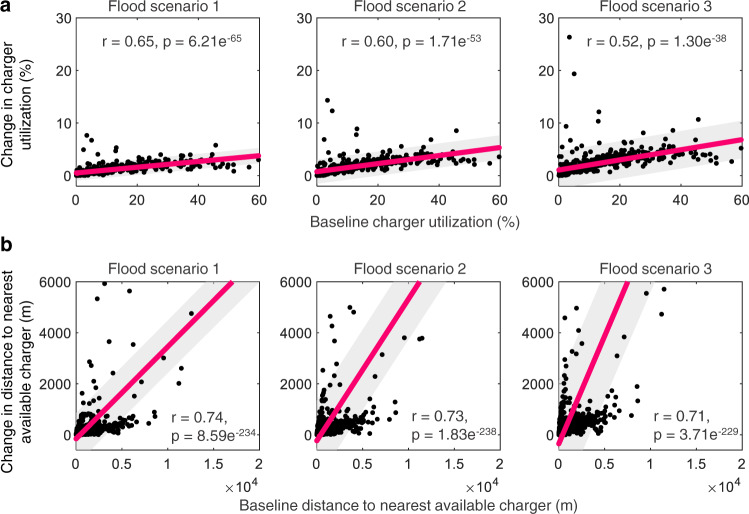
Where chargers are more stressed under flooding. **a** Correlation between the baseline charger utilization and the change in charger utilization due to flooding, only considering chargers where the latter was positive. The solid lines are the result of a linear regression, with the gray shaded areas representing the 95% confidence intervals. The corresponding Pearson’s correlation coefficient *r* and p-value are also indicated in each case. **b** The same as **a** but for the distance to the nearest available charger. The figures clearly show that flooding worsens the stress on already-stressed chargers.

Referring to Fig. 2, we see that the impact of flooding worsens with its intensity, as more chargers are taken out of service. Within the region at risk from flooding, flooded chargers experience a significant reduction in the utilization whereas those that remain available exhibit increases (see the peaks close to the *y*-axis in Fig. 2b). In the same region, for the charger accessibility, flooded chargers result in a negative impact due to the reduction in number of BEVs charging in that grid, whereas other grids experience positive impacts as users drive farther to access chargers. Beyond this region, the change in either metric is overwhelmingly positive, indicating that the chargers across Greater London are mostly additionally stressed. Observing the positively stressed grids (red plots) in Fig. 2b, d, we surprisingly find that in addition to the peak just outside the flooded region, there exists a significant peak farther away for each of the two metrics. For our simulations, the latter peak occurs ~10–13 km away, where, depending on the flood scenario, grids can experience a disproportionate increase of up to 50.9% in their charger utilization and BEV users walk an extra 269.9% from the nearest available charger to their intended destination. These increases are due to those users who no longer have access to chargers within the flooded areas and defer charging to later trips. Investigating the farther-located peaks in more detail, we find that, for each metric: (i) they occur at the same distance from the flooded areas as that of the corresponding baseline values, see Supplementary Note 1; and (ii) the magnitude of the change has a strong positive correlation with the baseline values of that metric, see Fig. 3 for the corresponding Pearson’s coefficients and *p* values. Furthermore, we find that the magnitude of the change in each grid is also positively correlated to the building density in that grid, which is closely related to the demand for EV chargers, see Supplementary Note 1. Clearly, these results imply that regions that are already stressed are the worst affected when flooding occurs, a trend that is independent of the region at risk from flooding, see Supplementary Note 2.

### Mitigating the impact of flooding

Our results have shown how flooding disperses the charging demand from the flooded areas into other parts of Greater London, resulting in disproportionate stresses on the rest of the charging network. Clearly, alleviating this stress requires placing new chargers outside the areas at risk from flooding. Here, we examine four potential strategies for siting these new chargers, developed based on our previous observations. In the first, we place additional chargers just outside the flooded region, ring-fencing the flooded area, in order to mitigate the stress on the immediate surroundings and potentially prevent its propagation into the remaining parts of the network. Next, given the strong correlation between the baseline usage levels of the chargers and the subsequent impact of a flood, the second strategy is usage-dependent placement, where we site the new chargers in grids with the highest baseline utilization (here, the top 2%). In the third, we place chargers near the peaks farther from the flooded regions in Fig. 2 with the aim of reducing them; we call this the distance-based strategy. Finally, in the fourth strategy, we distribute the new chargers randomly.

**Fig. 4:**
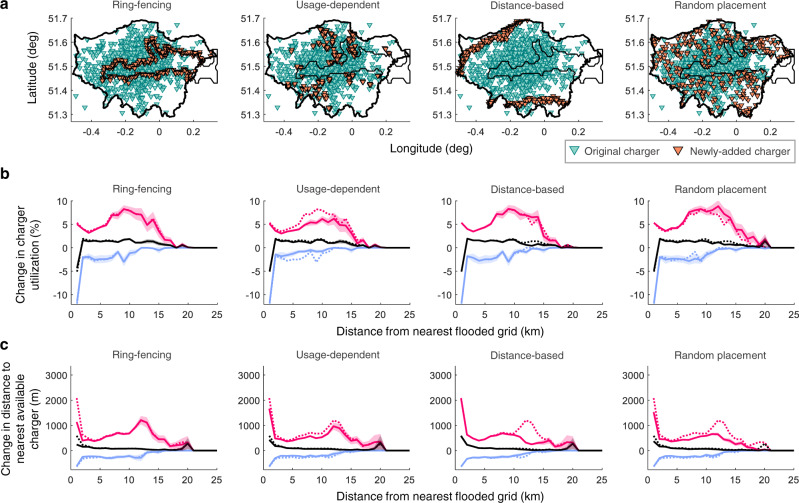
Mitigating the impact of flooding on charging infrastructure. **a** Locations of the existing and 5% additional chargers for each mitigation strategy. **b** How the change in charger utilization varies as a result of the mitigation strategy. The dotted lines represent the case with no mitigation, and are presented for comparison. **c** The same as **b** but for the distance to the nearest available charger. Results are only shown here for flood scenario-2; the corresponding plots for scenarios-1 and -3 are presented in Supplementary Note 3.

Implementing these and in each case adding 5% additional chargers, we present our results in Fig. 4. The corresponding plots when 2.5% and 10% additional chargers are added are presented in Supplementary Note 3. Broadly, we find that all the strategies increase access to chargers and improve user comfort, observing the reduction in the distance to the nearest available charger; see Fig. 4c. In more detail, the random placement strategy has the highest impact city-wide—achieving a peak reduction of 26.7% closest to the flooded region and 45.3% farther away—which is indeed expected as new chargers are introduced in all localities across the region. The usage-dependent strategy also has a city-wide, but smaller, impact. Meanwhile, ring-fencing and distance-based placement achieve targeted reductions in the peaks closest (35.9%) and farther away (42.8%) from the flooded region, respectively. However, they each have no significant impact on the other peak.

**Fig. 5:**
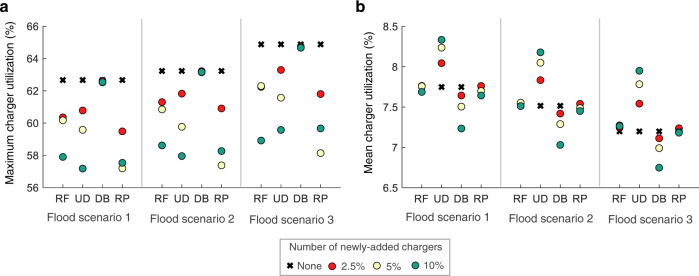
Charger utilization for the four mitigation strategies. **a** Maximum and **b** mean charger utilization values for ring-fencing (RF), usage-dependent (UD), distance-based (DB), and random placement (RP). Results are shown when the number of added chargers varies as 2.5%, 5%, and 10%.

In terms of mitigating the impact of flooding on charger utilization, the usage-dependent strategy exhibits the best performance, resulting in the highest reduction of 24.4% in the peak farther from the flooded area, see Fig. 4b. This observation may be explained by the strong positive correlation between the baseline utilization and the flood impact; improving the availability of chargers in regions with high demand reduces the baseline utilizations and thereby the flood impact. Ring-fencing, on the other hand, affects only grids in the near-vicinity of the flooded areas, reducing their peak utilization by 5.7%. For the other strategies, we observe no immediately apparent improvement; this does not however mean that these are completely ineffective in reducing utilization-related stress. Referring to Fig. 5 which presents the peak and mean utilization values considering all chargers in the network by removing the distance dimension, we find that the peak reduces regardless of the strategy chosen, signifying reduced stress on the network. Here, random placement and ring-fencing show comparable performances with that of the usage-dependent strategy, given that some new charger sites happen to overlap with the stressed areas (see Fig. 4a). Finally, despite performing the best in improving the charger accessibility farther away from the flooded region, the distance-based strategy performs the worst in terms of the chargers’ utilization, with at most 0.3% reduction of the peak with even 10% additional chargers. This is likely as the newly added chargers do not overlap appreciably with the stressed areas. An interesting point is that the usage-dependent strategy differs from the rest in that it increases the mean charger utilization (see Fig. 5b). With the peak value reducing, this implies that the augmented charger network is more efficiently used.

## Discussion

While our simulations of the present-day charging network in Greater London did not yield a peak utilization of 100% on any part of the network due to flooding, such a situation could occur if BEV growth outpaces the expansion of the charging infrastructure. Given that the disruption is most concentrated on already-stressed parts of the network and its repetitive nature, users who will regularly depend on chargers in the particularly affected parts may find it challenging to find available chargers and therefore be discouraged from adopting or continuing using BEVs over time. Arguably, we cannot avoid placing chargers in areas that are at risk from flooding, as this would inconvenience riders under normal circumstances. As we see in the case of Greater London, this region includes both commercial areas that serve as retail and entertainment centers, and residential neighborhoods. Therefore, city-specific flood impact analyses must be performed and mitigation plans drawn up, perhaps by combining the best aspects of the different mitigation strategies that we have studied here. If the region at risk from flooding changes over time, e.g., due to new land developments, additional flood prevention infrastructure, or climate change, the analysis presented here could be reexamined considering all the chargers in the network existing at that point in time.

Below, we provide specific recommendations for policymakers in Greater London on including flood resilience as an additional consideration while planning new investments into the EV charging infrastructure. First, we have shown that the usage-dependent strategy performs the best in terms of improving the flood resilience, accessibility, and efficiency of the charger network. For this, our results show that around 5–10% additional chargers are needed to restore the peak charger utilization to baseline values when flooding occurs (see Table 1 and Fig. 5). With the majority of new public chargers in Greater London slated to be installed by the private sector^44^, the self-incentivizing nature of the strategy will ensure profitability and foster competition between service providers. Importantly, the support infrastructure, e.g., electricity distribution systems and transformers, must also be upgraded as necessary. Second, given that the areas around the Thames are the ones vulnerable to flooding, the immediate impact will be felt on chargers in central London; ring-fencing this area with additional chargers irrespective of the baseline demand will reduce the peak stresses and improve accessibility in a targeted manner. Finally, the random placement strategy will improve accessibility at the edges of Greater London, particularly to the North-West, East and South. We note here that while some of the charger sites may not be profitable for the ring-fencing and random placement strategies, the government may intervene to subsidize these installations to improve charger accessibility. As Transport for London notes^44^, government support is key to ensuring consumer confidence and accelerating EV adoption.

Here, we present a few remarks on the approach we adopt for our study. First, we do not consider how flooding events alter the number of BEV rides and or their destinations. This is a reasonable assumption in situations where drivers only regard the flood as an inconvenience rather than a deterrent to travel. Second, we do not consider dynamic changes in the location and impact of floods, assuming that any chargers that are flooded remain out of service throughout the day. Third, we assume no interoperability issues between BEVs and chargers, i.e., any BEV can charge at any charger. Finally, our study proposes four qualitative approaches to mitigating the impact of flooding on the EV charging infrastructure. Given more high-resolution city-specific data, e.g., pertaining to installation costs, revenue, and flood risk, policymakers may augment these approaches with multi-criteria optimization methods to determine where the new chargers can be best sited.

## Methods

### Building and road network specifications

We obtained building and road network data for Greater London from OpenStreetMap (OSM)^45^. In more detail, we began by obtaining all nodes and ways from OSM within the region—defined by the OSM relation 175342^46^—that represent buildings. This .osm data file, obtained through Overpass turbo^47^, was converted into a MATLAB structure using functions from ref. ^48^. In addition to buildings’ locations, we obtained information about their type, i.e., whether they are residential (identified by the value of the key “building” being equal to “residential”), or commercial (the value of the key “building” equal to “commercial”). This information is utilized while determining the destinations of the BEV rides in our simulations, depending on their trip purposes. Here, due to the crowd-sourced nature of OSM, several buildings did not have a classification, and were designated randomly as residential, work, and commercial with probabilities 90%, 5%, and 5%, respectively. Note that buildings with a “commercial” classification are considered to be non-work-related. Further, we found that buildings already classified as “commercial” were spread out throughout the Greater London area^49^, which justifies the random classification of the unclassified buildings. As for the road network, we obtained the set of OSM nodes and ways such that the value of the ways’ “highway” key equals one of the following: “motorway”, “trunk”, “primary”, “secondary”, “tertiary”, “unclassified”, “residential”, or “service”. Using the code described in ref. ^50^, we first merged nodes within 30 m of each other to form a single node, to reduce the computational complexity. We then improved the connectivity of the road network (the OSM network may not be fully connected due to its crowd-sourced nature) by creating new edges between any strongly connected sub-networks until all nodes were strongly connected. Finally, the road network was contracted to remove any edge that had exactly one predecessor and one successor, or had exactly two predecessors that were also its only two successors. Each building was assumed to be connected to the node on the road network that is closest to it.

### EV charging infrastructure

The locations of the public EV chargers in Greater London were taken from the website maintained by the Office of the Mayor of London^51^, which consists of the locations of slow (defined as < 43 kW) and rapid chargers (defined as ≥43 kW). Overall, we obtained data for 5925 chargers across the region as a .csv file from the ArcGIS platform, see Supplementary Note 4. Each charger was assumed to be connected to the node on the road network that is the closest to its location. According to a report from the International Council on Clean Transportation^30^, more than 40% of drivers in Greater London do not have off-street parking. Accordingly, we assume that 25% of BEVs each depend on public night-time charging and daytime workplace/commercial charging, and the rest 50% depend on residential charging. Since the actual charging power depends on the model of the BEV and the charger, for simplicity in our simulations, the slow chargers are assigned a power of 12.5 kW (the average of the reported lower and upper limits, respectively, 3 kW and 22 kW) and the rapid chargers, 43 kW, corresponding to the definitions from ref. ^30^. All the chargers added to mitigate the impact of flooding are considered to be rapid chargers to allow us to assess the best-possible outcome. We further assume 89% efficiency for all the chargers, similar to the approach adopted in ref. ^1^.

### Simulating rides

We simulated personal electric vehicle trips using MATLAB; the overall flowchart is shown in Supplementary Note 5. We selected the number of EVs to be between 6 and 8 times that of the chargers, based on the 2020 statistics for Greater London^30^. Results presented in the main article correspond to the value 6, whereas Supplementary Note 1 presents results for the value 8. Given that the share of BEVs over all EVs (which also includes hybrid EVs) is projected to reach 90% by 2025 and 100% by 2030^30^, we assumed that the entire set of EVs in our simulations are comprised of BEVs. We consider three models of BEVs: Nissan Leaf (40 kWh battery), Nissan Leaf Plus (62 kWh battery), and Tesla Model S (100 kWh battery). In each simulation, the vehicles are assigned with equal probability one of these models.

The BEV trips were simulated according to the travel patterns reported by Transport for London^52^. This data pertains to a survey conducted until 2011, and reports details including the number, duration, and distribution of trips for the residents of Greater London. Vehicles begin and end at home (or a parking spot close to home), and the entire journey over 24 h is referred to as a tour. Each vehicle can make two or more trips in their tour, defined as starting from one location in Greater London and ending at another, during the day. The number of trips for each BEV is determined using the probabilities presented in Supplementary Table 1 in Supplementary Note 6. In our study, the average number of trips per BEV is 2.42, which matches closely with the surveyed data from ref. ^52^ which is 2.49. While the final trips for the BEVs terminate at home, the interim destinations in each BEV’s schedule are determined based on the purpose of each trip. Since particular destination locations are not specified in the survey, we selected them randomly across the region using available trip-purpose data, from the appropriate building type: (i) work or (ii) commercial (shopping, public spaces, leisure, escort or school); the probabilities for each are shown in Supplementary Table 2 in Supplementary Note 6. A previous study^53^ of EV users in the UK has shown that there is no seasonality in the charging behaviors. We therefore only consider trips on a typical weekday, and the probability distribution of the departure times of the BEVs are given in Supplementary Fig. 22 in Supplementary Note 6. The speed of vehicles through the day is taken from the Transport for London report^54^, within the ranges presented in Supplementary Table 3 in Supplementary Note 6. At each instant of the simulation, the average speeds of the vehicles are selected randomly between the minimum and maximum values specified. Based on these speed values, the arrival times of the BEVs are estimated for each trip, assuming that they traverse the shortest distance between the source and the destination. Overall, the average Haversine distance of a trip in our simulations is 15.8 km, which corresponds well to the value of 13.9 km obtained in prior surveys^52,55^. The BEVs begin with state-of-charge (SOC) values randomly selected in the interval [*a*, *b*], which depends on whether the BEV relies on residential charging or night-time public chargers close to home (*a* = 0.9, *b* = 1.0), or on public chargers during the day (*a* = 0.4, *b* = 0.6). The SOC of the *i*th BEV at the end of a trip of distance *d*_*trip*_ is calculated as follows:

1 $${{{{{{{{\rm{SOC}}}}}}}}}_{i,dest}={{{{{{{{\rm{SOC}}}}}}}}}_{i,source}-{{{{{{{{\mathcal{D}}}}}}}}}_{i}\cdot{d}_{trip}$$

where the discharge rate ${{{{{{{{\mathcal{D}}}}}}}}}_{i}$ depends on the BEV model, see Supplementary Note 6.

The simulation of EV charger usage for 24 h is carried out using simulations with 1-min resolution. At every time step, an event-triggered algorithm generates arrival and departure events based on the estimated arrival and departure times for each trip.

#### Arrival event

A user decides to charge their BEV after a trip if the SOC falls below a threshold *λ*_1_ (taken here as 0.5). The user then drives to a charger that is closest to the destination and currently available, considering an upper limit on the distance between the destination and the charger to be 300 m. If the SOC falls below a lower and more critical threshold *λ*_2_ (taken here as 0.3), the user seeks the nearest available charger at any distance from the destination. The assignment of the chargers to the BEVs is carried out in a first-come-first-served basis. Charging only occurs if there remain at least 30 min to the departure time for the next trip in the BEV’s schedule.

#### Departure event

If a BEV charges at the completion of a trip, the charger’s location is updated as the actual location in the trip schedule instead of the intended destination; the difference between the two is noted as the ‘distance to the nearest available charger’ for that trip. During the charging process, the SOC of the BEV changes as follows:

2 $${{{{{{{{\rm{SOC}}}}}}}}}_{i,end}={{{{{{{{\rm{SOC}}}}}}}}}_{i,start}+{{{{{{{{\mathcal{C}}}}}}}}}_{i}\cdot{T}_{wait}$$

where *T*_*wait*_ is the total charging time, and ${{{{{{{{\mathcal{C}}}}}}}}}_{i}$, the charging rate; see Supplementary Note 6. The trip distance and actual arrival time are determined from the shortest distance from the origin of the trip to the destination. When a charging BEV departs, it releases that charger to be used by others.

Overall, a BEV is considered to have failed if its SOC falls below 0.2 at any time^1^. In addition, a BEV dependent on day-time public charging is considered to have failed if its SOC at the end of the day falls below 0.3, considering that the user must then drive to a nearby charger on the following day to charge.

### Flooding data

To obtain the regions in Greater London that are at risk from flooding, we used the tool developed by Climate Central^43^, which projects the land area under a given flood level considering sea level rise and coastal flooding. The following settings were used: Projection Type = sea level rise + moderate flood; Pollution Pathway = current trajectory; Luck = medium; Areas to show as threatened = exclude areas isolated by higher land; and Sea-level-projection source = leading Consensus (IPCC 2021). Specifically, we used projections for the year 2030, which is the closest available data point. Notably, other estimates of flood risk in Greater London, such as from the UK Environment Agency^56^, also present very similar results when other sources of flooding (river and surface water) and existing flood defenses are included, see Supplementary Note 7. The Climate Central data corresponds to a 10% risk per year over these areas, while the UK Environment Agency data projects anywhere from greater than 0.1% to greater than 3.3% per year.

We obtained the regions at risk in Greater London as a .png map image and utilized an open source tool^57^ to extract the coordinates of the regions at risk. Subsequently, we overlaid for simplicity a 42 × 86 grid on the geographical area of Greater London between the latitudes 51.280 and 51.692, and the longitudes −0.510 and 0.340. All grids which overlapped with the at-risk regions were considered to be at risk from flooding; we assume that the flooded grids do not change during the day. For each simulation, the public chargers within each at-risk grid are assumed to become unavailable for use with the probability *p*_*f*_, which is selected as 0.5, 0.7, and 0.9, and designated as flood scenarios-1, -2, and -3, respectively. We note that BEVs that depend on night-time public chargers within a flooded grid are assigned lower SOC values at the beginning of the day, in the range [0.4, 0.6], to reflect their inability to charge.

## Supplementary information

Supplementary Materials

## Data Availability

The electric vehicle charger, road network, and building data used in this study are available at ref. ^58^.

## Code availability

Codes for the simulations in this study are available at ref. ^58^.
