# Supplementary material for: Resilience of urban public electric vehicle charging infrastructure to flooding
Source: Nat Commun. 2022 Jun 9;13:3213. doi: 10.1038/s41467-022-30848-w (PMC9184540; doi:10.1038/s41467-022-30848-w)
Supplement: Supplementary file 1 — Supplementary Materials [file 41467_2022_30848_MOESM1_ESM.pdf]

Supplementary Materials for

Resilience of urban public electric vehicle charging infrastructure to flooding

Gururaghav Raman[†], Gurupraanesh Raman[†], and Jimmy Chih-Hsien Peng[*]

[*] Corresponding author. E-mail: jpeng@nus.edu.sg
[†] These authors contributed equally: Gururaghav Raman, Gurupraanesh Raman

This document is structured as follows:

- **Supplementary Note 1** (*page 2*) presents additional results demonstrating the impact of flooding on BEVs and charging infrastructure;
- **Supplementary Note 2** (*page 6*) presents simulation results when the region at risk from flooding is varied across Greater London;
- **Supplementary Note 3** (*page 14*) presents additional results demonstrating the impact of the four mitigation strategies presented in the main article;
- **Supplementary Note 4** (*page 18*) presents the locations of the public EV chargers in Greater London used in our simulations;
- **Supplementary Note 5** (*page 19*) presents a flowchart of the simulation methodology adopted in our study;
- **Supplementary Note 6** (*page 20*) presents additional data that we used to simulate BEV rides; and
- **Supplementary Note 7** (*page 22*) presents a comparison of the regions at risk from flooding in Greater London indicated by different sources.

1 Supplementary note 1: Impact of flooding

1.1 Impact of flooding on BEV success

This subsection presents results regarding the impact of flood events on the success of BEV rides; see Supplementary Figure 1.

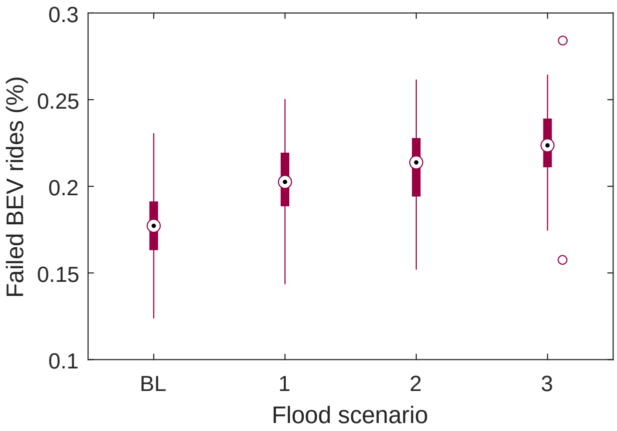

Supplementary Figure 1. Failure rate of BEV rides for different flood scenarios, over 100 simulations. The central mark, bottom, and top edges of each box plot represent the median, 25th, and 75th percentiles, respectively. Outliers, if any, are marked as individual circles. [BL: Baseline scenario with no flooding]

1.2 Geospatial analysis of the impact of flooding

Here, we first demonstrate that the peak change due to flooding in the two metrics considered: (i) charger utilization, and (ii) distance to the nearest available charger, occurs at around the same distance where the respective baseline value also peaks. This is shown in Supplementary Figure 2.

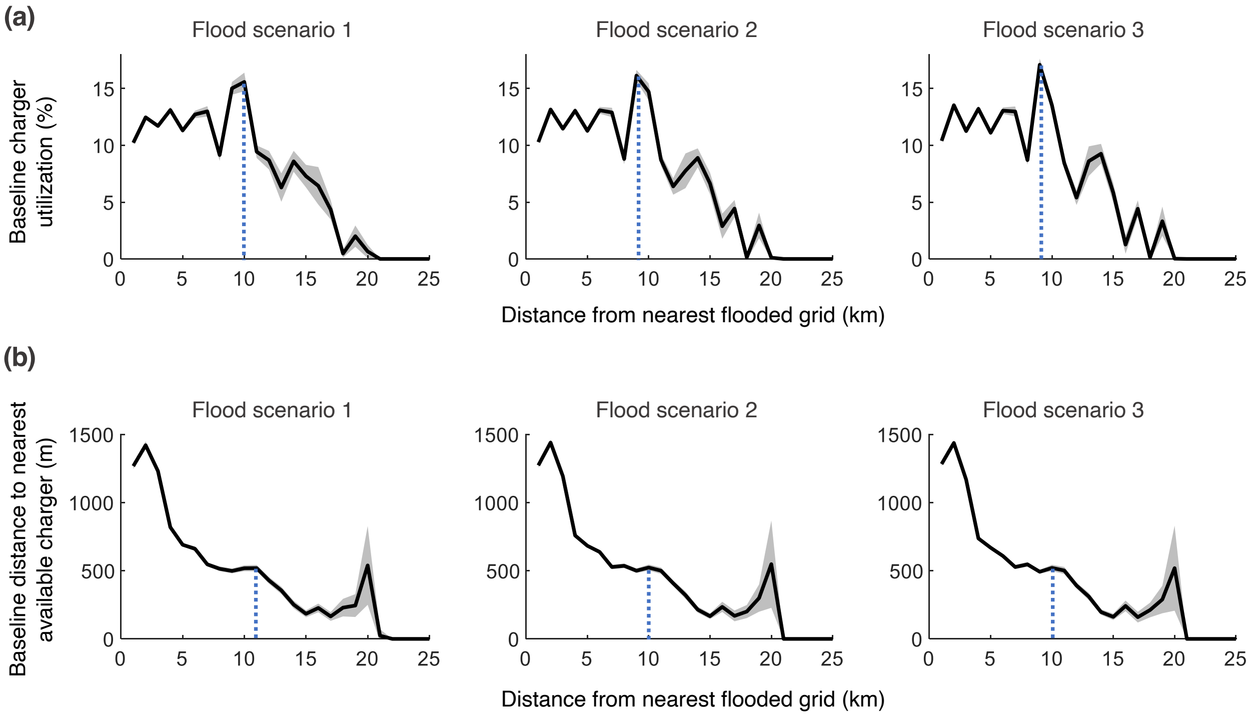

Supplementary Figure 2. Plot of (a) the grid-wise average baseline charger utilization and (b) the distance to the nearest available charger, summed grid-wise, with the distance from the nearest flooded grid. Results are presented as an average over 100 simulations, with the shaded areas representing the 95% confidence intervals.

In Fig. 3 in the main article, we observe a strong positive correlation between the magnitude of the change in the two metrics for different regions in Greater London with their respective baseline values. Here, we study the underlying geospatial factors in more detail, which are (i) the building density, and (ii) the charger density. We define these terms respectively as the number of buildings and chargers within each grid; they are shown in Supplementary Figure 3.

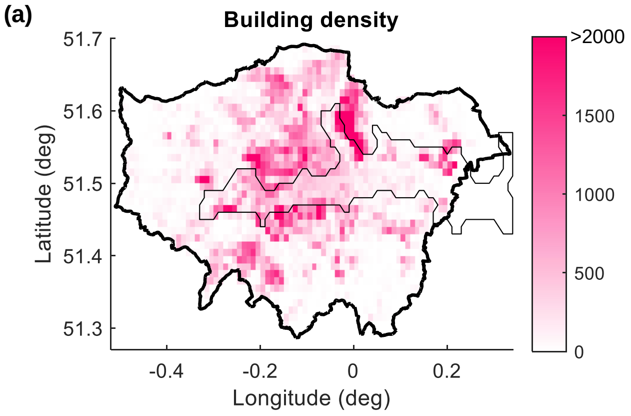

Supplementary Figure 3. (a) Grid-wise building density and (b) grid-wise EV charger density in Greater London. The region at risk from flooding is also outlined for context.

We subsequently assess the correlation between both the building and charger densities, and the impact of flooding. The results of this analysis are shown in Supplementary Figure 4. The following can be observed:

1. The positive, statistically significant correlations in Supplementary Figure 4(a) and (b) imply that in general, areas with a higher building density experience a larger increase in the two metrics as a result of flooding. This is also visually evident from comparing Supplementary Figure 3(a) with Figs. 2(a) and (c) in the main article.

2. As for charger density, we find small negative, statistically significant correlations in Supplementary Figure 4(c). Intuitively, the higher the charger density, the more the number of chargers that are present to absorb the increase in arriving BEVs that are prevented from charging earlier due to the flood. Vice versa, for a grid with a smaller charger density, the average utilization could increase significantly when a small number of chargers experience an increased consumption. As such, this observation supports our solution approach of introducing additional chargers to mitigate the impact caused by flooding.

3. No statistically significant correlations can be observed in Supplementary Figure 4(d), suggesting no obvious relationships exist between the change in the distance to the nearest available charger and charger density. This may potentially be explained due to two conflicting phenomena: when charger density is high for a grid, (i) more users may show up, thereby contributing to the value of the total distance walked by users from their intended destinations to nearby available chargers; or (ii) more chargers may be available closer to users' intended destinations, reducing the overall distance walked.

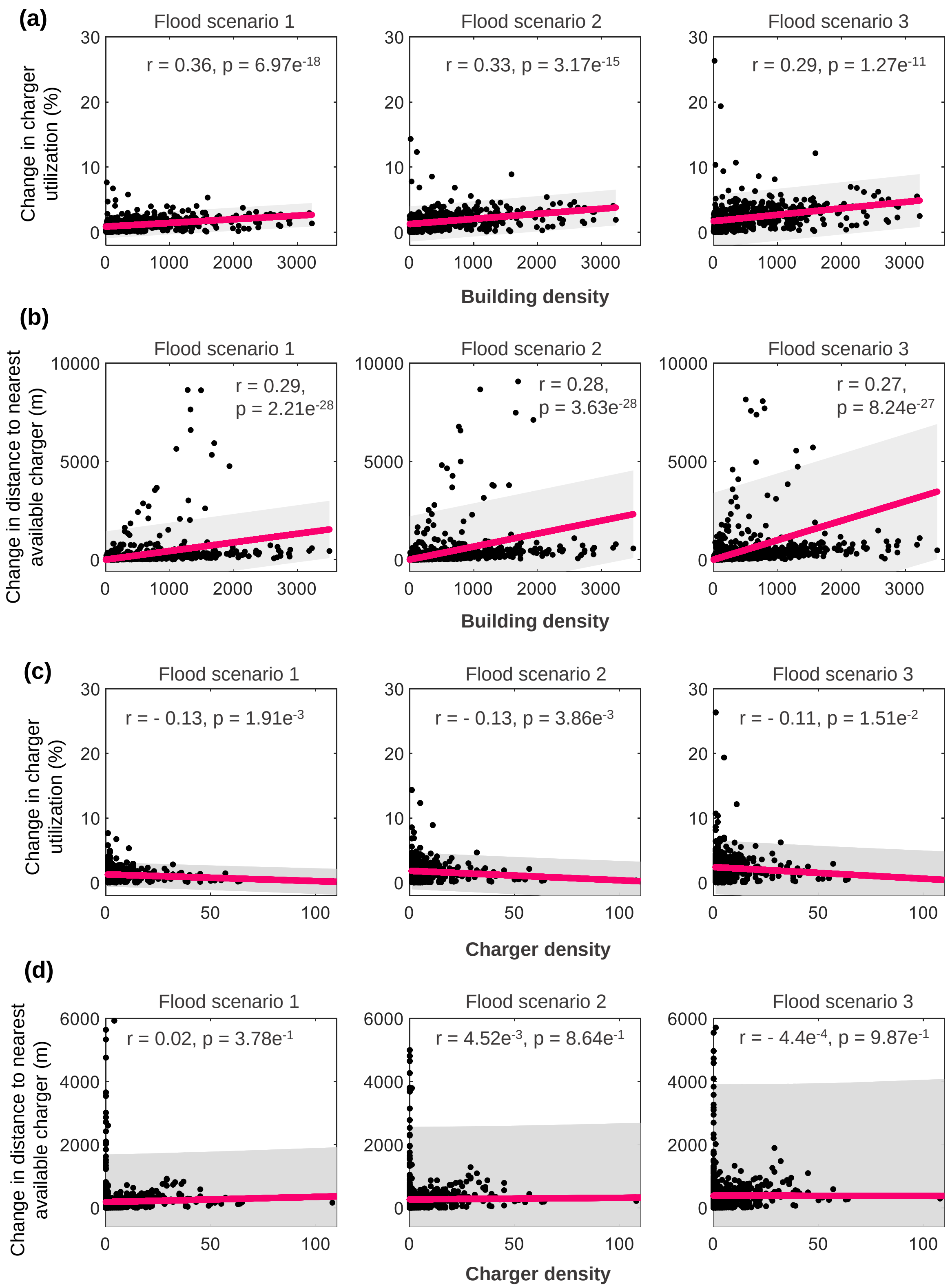

Supplementary Figure 4. Correlations of the geospatial parameters: grid-wise building density and grid-wise EV charger density, and the impact of flooding measured by the change in the charger utilization and the distance to the nearest available charger. We only consider locations where the respective metrics increased as a result of flooding. The solid lines are the result of a linear regression, with the grey shaded areas representing the 95% confidence intervals. The corresponding Pearson's correlation coefficient r and p-value are also indicated in each case.

1.3 Varying the number of BEVs per charger

Supplementary Figure 5 illustrates how the impact of a flooding event varies when the number of BEVs per public charger changes.

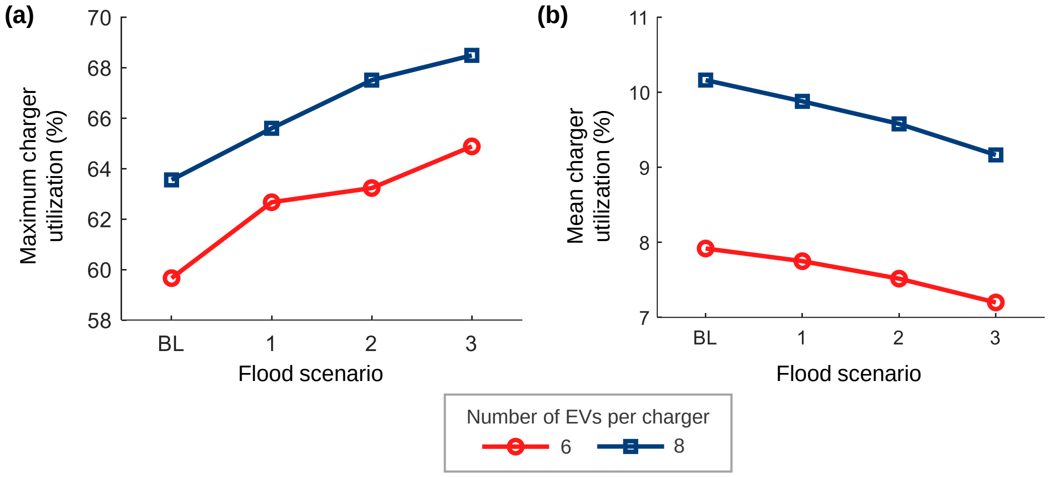

Supplementary Figure 5. How the maximum and mean utilization level of the chargers in Greater London change when the number of BEVs per public charger is changed. Results are presented as an average over 100 simulations. [BL: Baseline scenario with no flooding]

2 Supplementary note 2: Varying the regions at risk from flooding

To understand how our simulation results vary when the regions that are at risk from flooding change, we consider five different hypothetical cases, see Supplementary Figure 6. Similar to our analyses in the main article, we consider three scenarios that reflect progressively increasing intensity of flooding:

- Flood scenario 1: An at-risk grid has a probability $p = 0.5$ of being flooded.
- Flood scenario 2: An at-risk grid has a probability $p = 0.7$ of being flooded.
- Flood scenario 3: An at-risk grid has a probability $p = 0.9$ of being flooded.

For the five hypothetical cases, Supplementary Figure 7 presents the number of chargers flooded for the different flood scenarios.

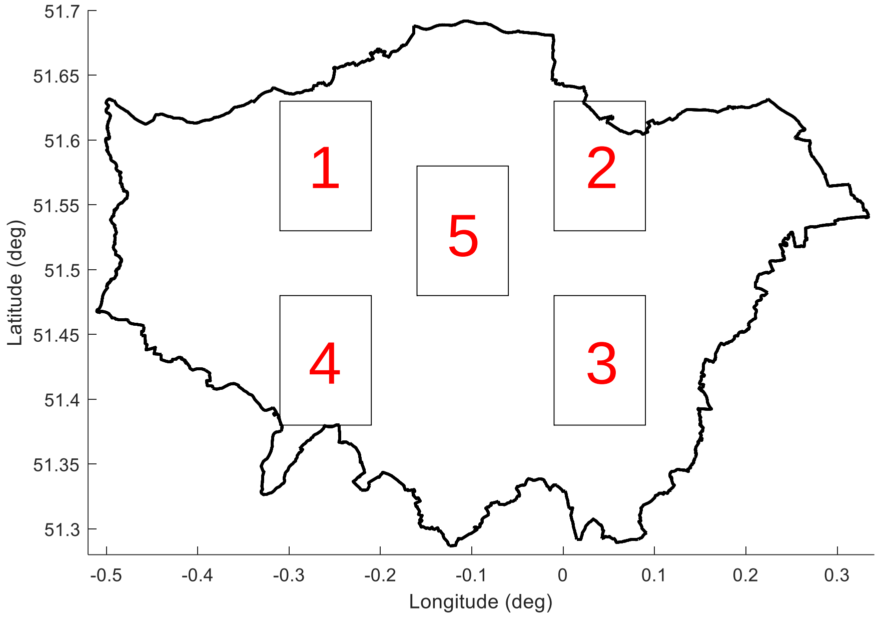

Supplementary Figure 6. Regions at risk from flooding for five hypothetical cases.

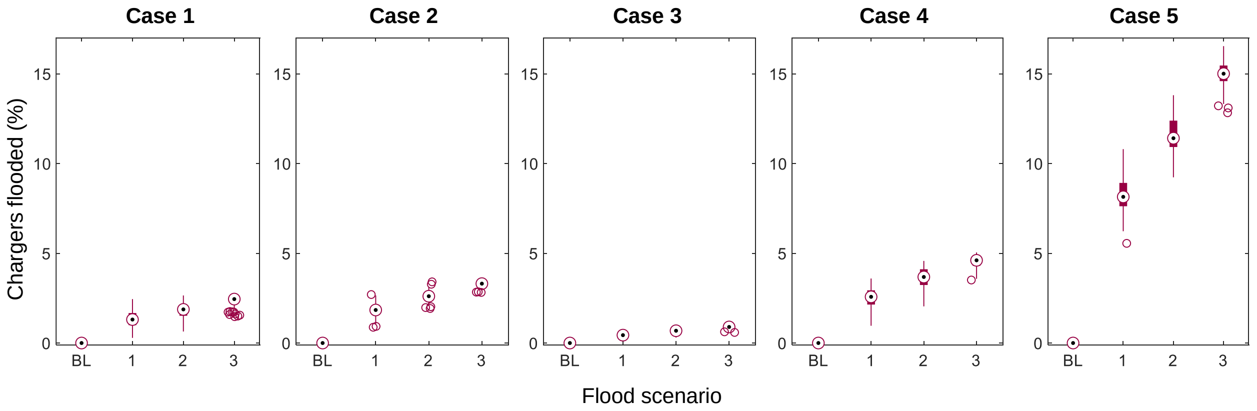

Supplementary Figure 7. Number of chargers flooded. Results are presented as an average over 100 simulations, in each of which the grids flooded are determined randomly based on the flooding probabilities. The central mark, bottom, and top edges of each box plot represent the median, 25th, and 75th percentiles, respectively. Outliers, if any, are marked as individual circles. [BL: Baseline scenario with no flooding]

The results of our simulations are presented in Supplementary Figures 8– 12, shown over 100 simulations, in each of which the grids flooded are determined randomly based on the flooding probabilities.

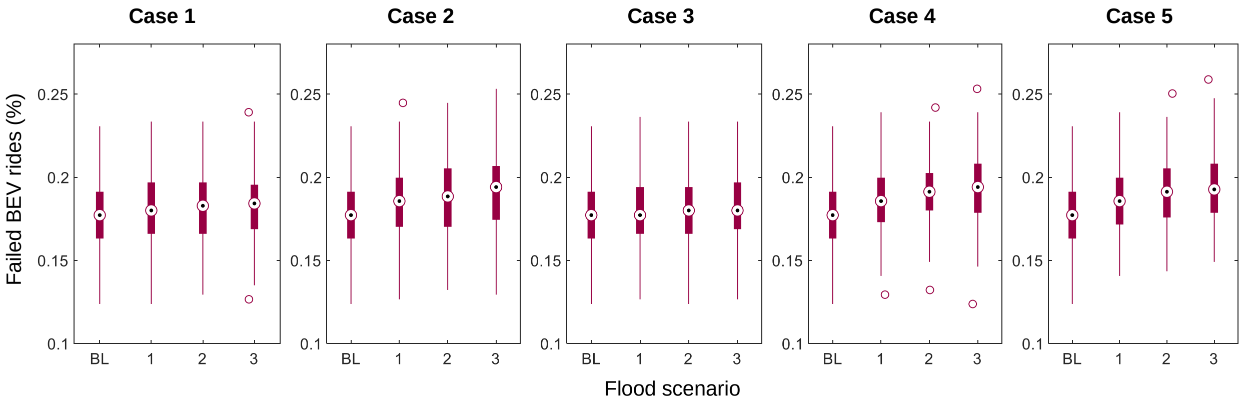

Supplementary Figure 8. Failure rate of BEV rides for different flood scenarios. The central mark, bottom, and top edges of each box plot represent the median, 25th, and 75th percentiles, respectively. Outliers, if any, are marked as individual circles. [BL: Baseline scenario with no flooding]

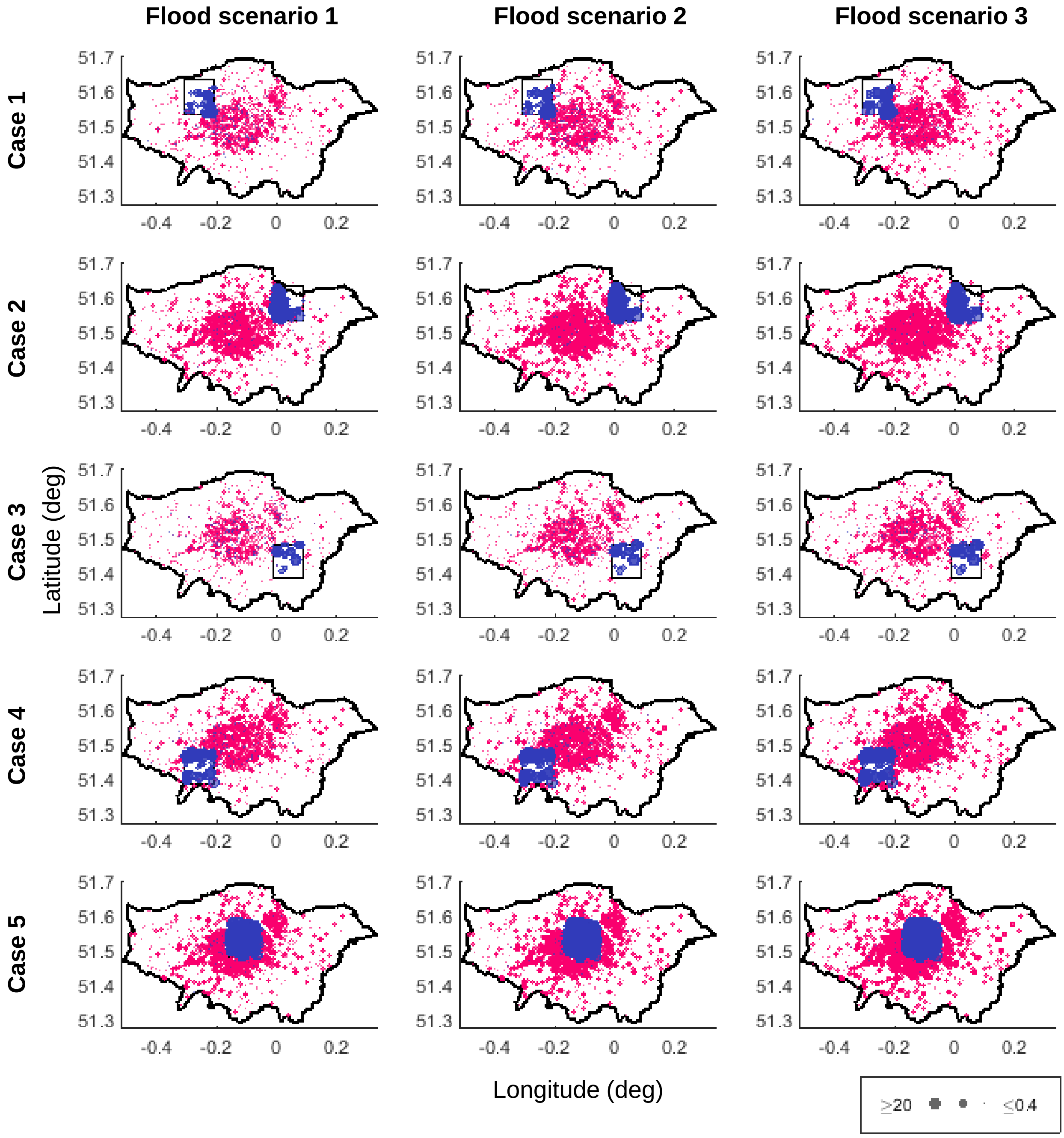

Supplementary Figure 9. Change in charger utilization (time used per day (%)): Same as Fig. 2(a) in the main article, but for the five hypothetical cases presented in Supplementary Figure 6.

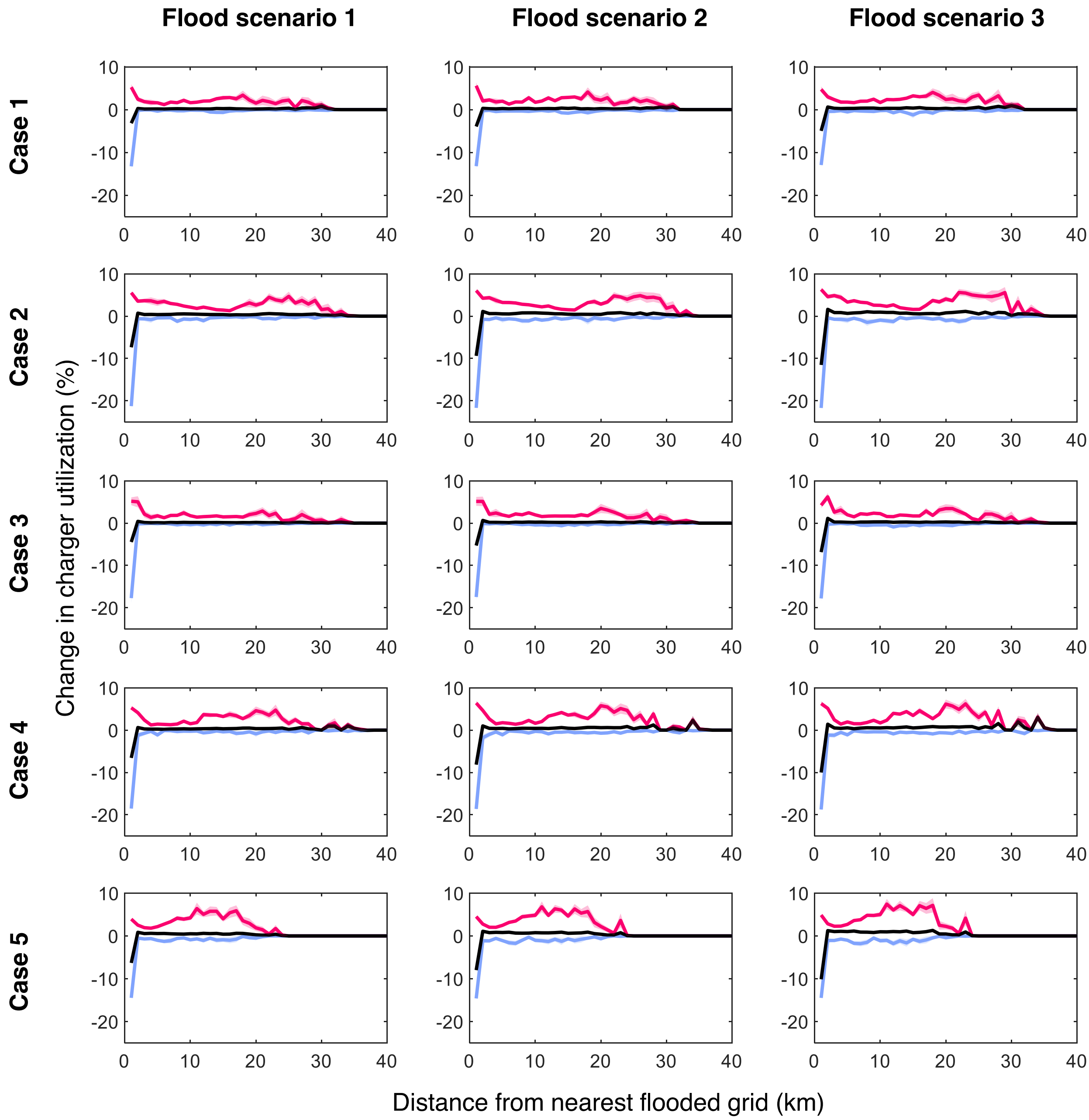

Supplementary Figure 10. Same as Fig. 2(b) in the main article, but for the five hypothetical cases presented in Supplementary Figure 6.

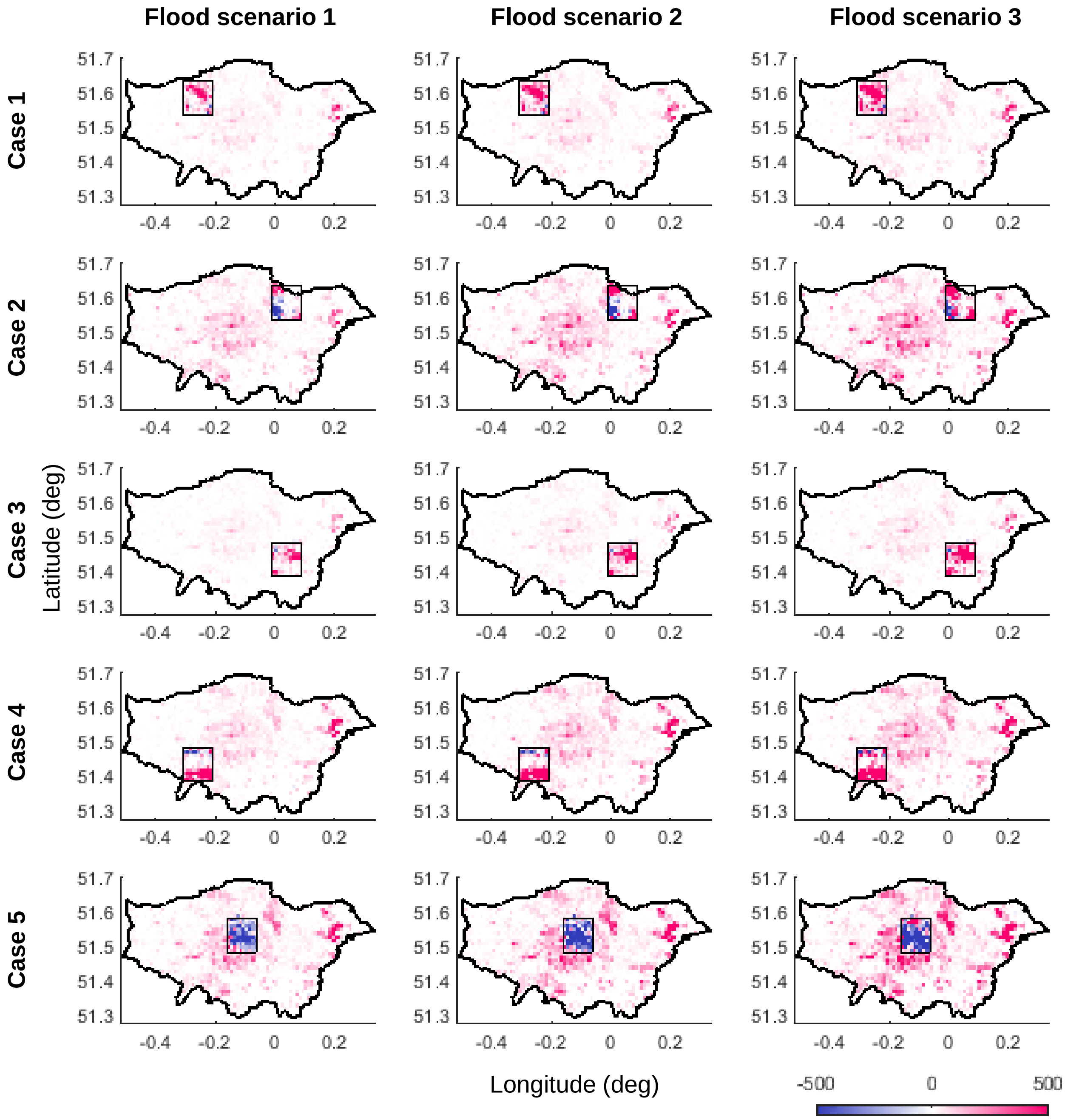

Supplementary Figure 11. Change in the distance to the nearest available charger (meter): Same as Fig. 2(c) in the main article, but for the five hypothetical cases presented in Supplementary Figure 6.

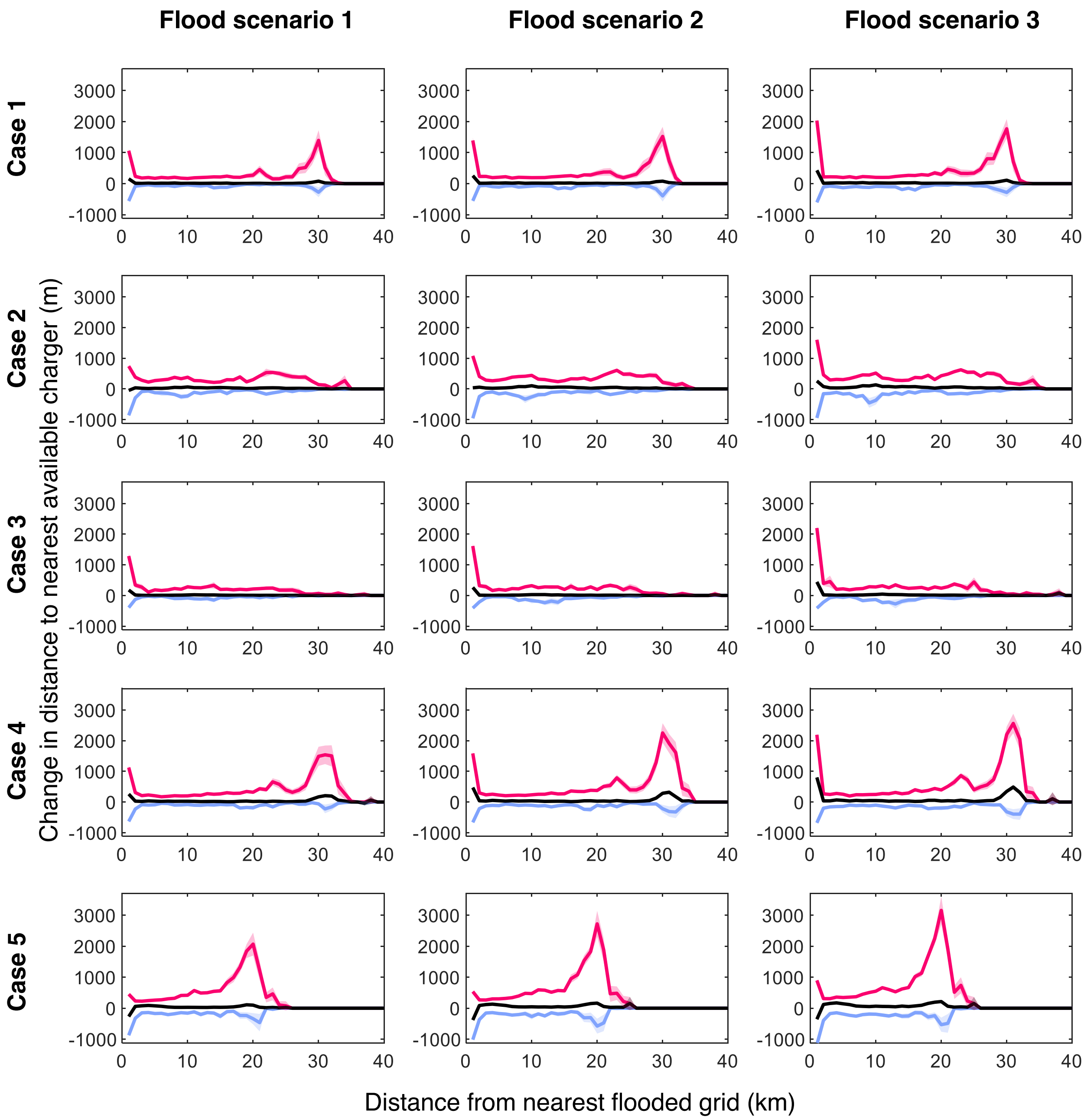

Supplementary Figure 12. Same as Fig. 2(d) in the main article, but for the five hypothetical cases presented in Supplementary Figure 6.

2.1 Geographic correlation between baseline metrics and the impact of flooding

This subsection demonstrates that the peak change due to flooding in the two metrics considered: (i) charger utilization, and (ii) distance to the nearest available charger occurs at around the same distance where the respective baseline value also peaks. Clearly, this trend—also visible in Supplementary Figure 2 for the actual regions at risk from flooding in Greater London that are considered in the main article—is independent of the region at risk from flooding.

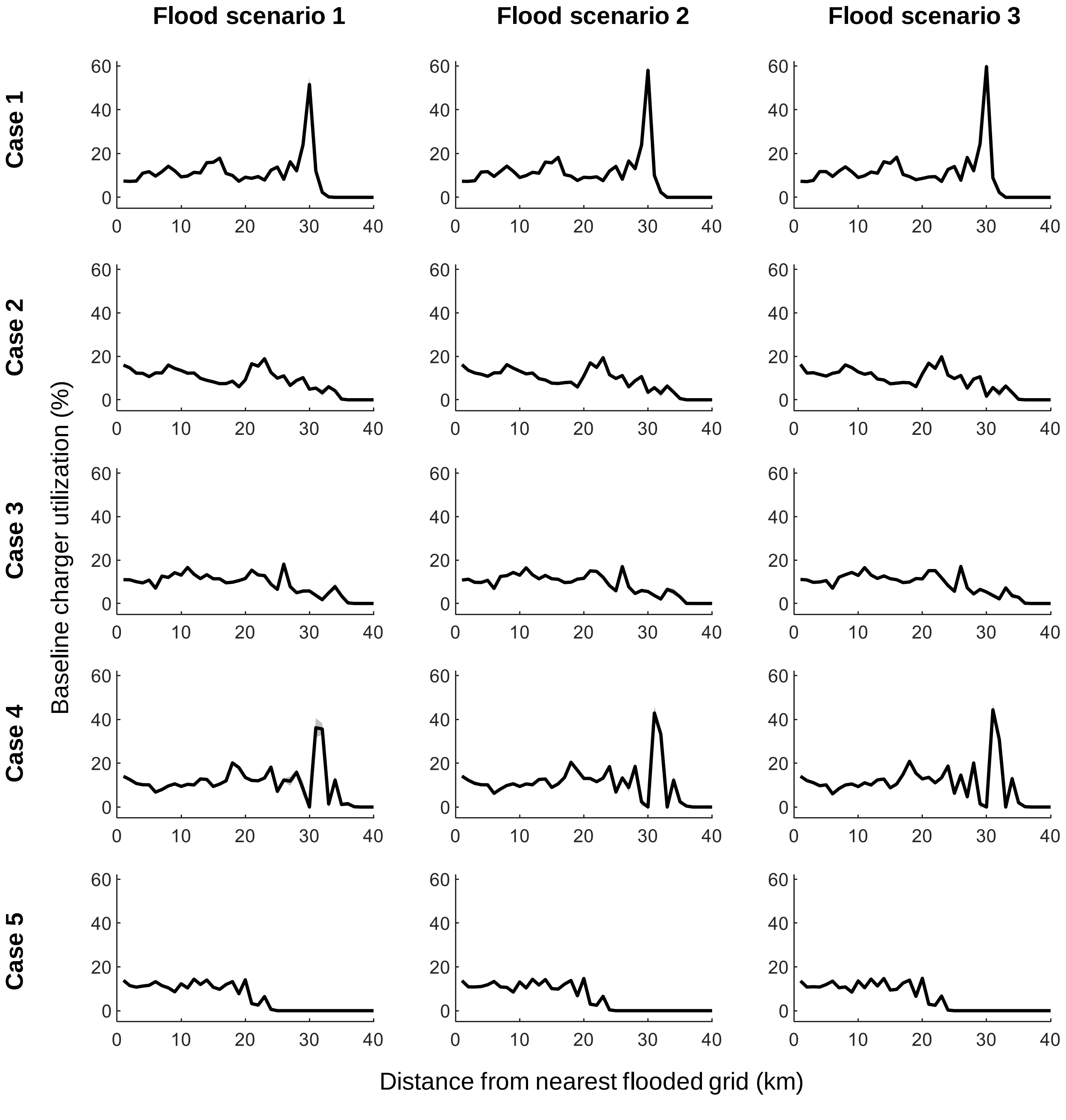

Supplementary Figure 13. Plot of the grid-wise baseline charger utilization with the distance from the nearest flooded grid, for the five hypothetical cases presented in Supplementary Figure 6.

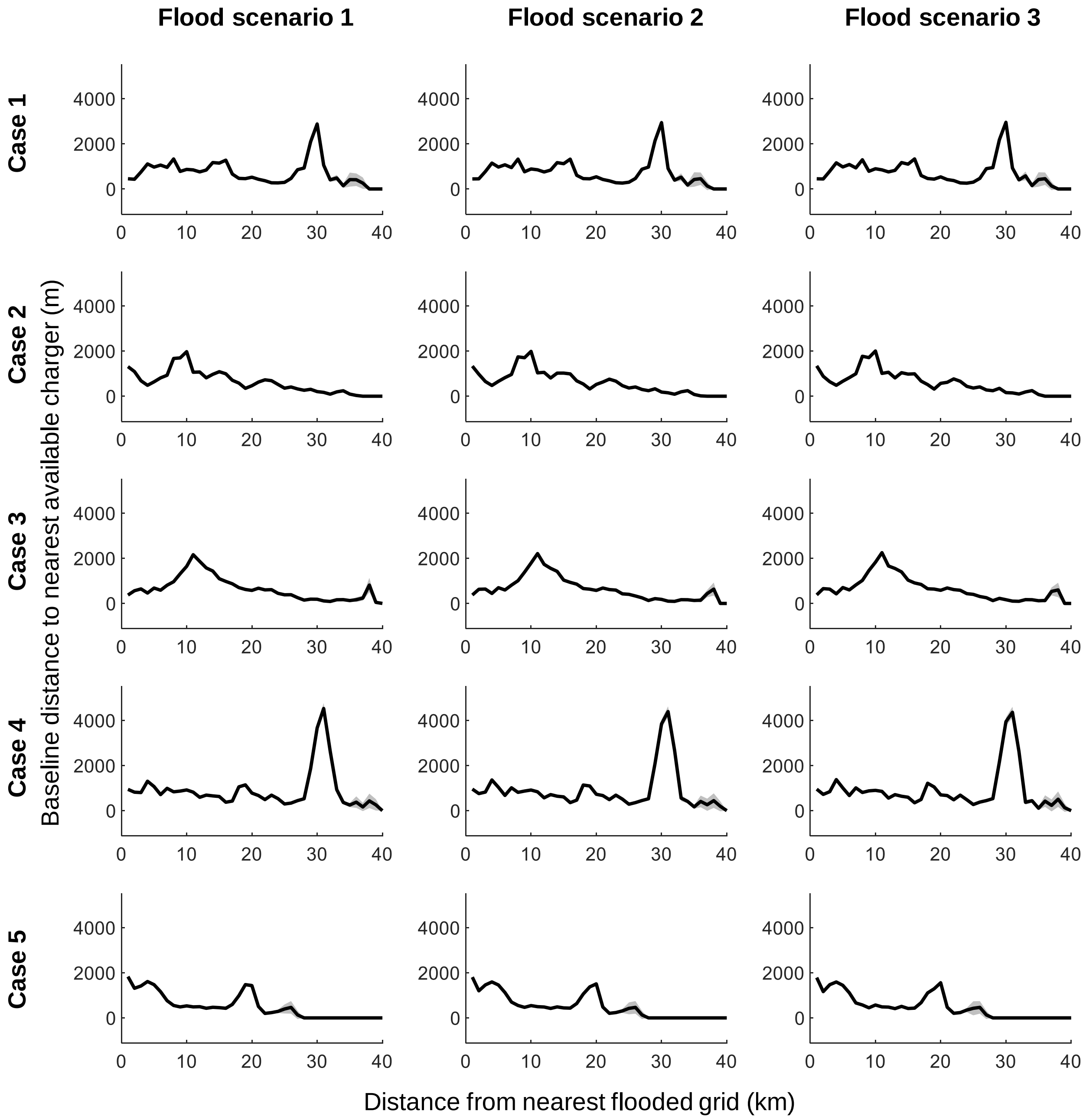

Supplementary Figure 14. Plot of the baseline distance to the nearest available charger, summed grid-wise, with the distance from the nearest flooded grid, for the five hypothetical cases presented in Supplementary Figure 6.

3 Supplementary note 3: Mitigating the impact of flooding

3.1 Impact on BEV success

Supplementary Figure 15 presents how the results shown in Supplementary Figure 1 change when four different mitigation strategies are implemented. In each case, 5% additional chargers are added.

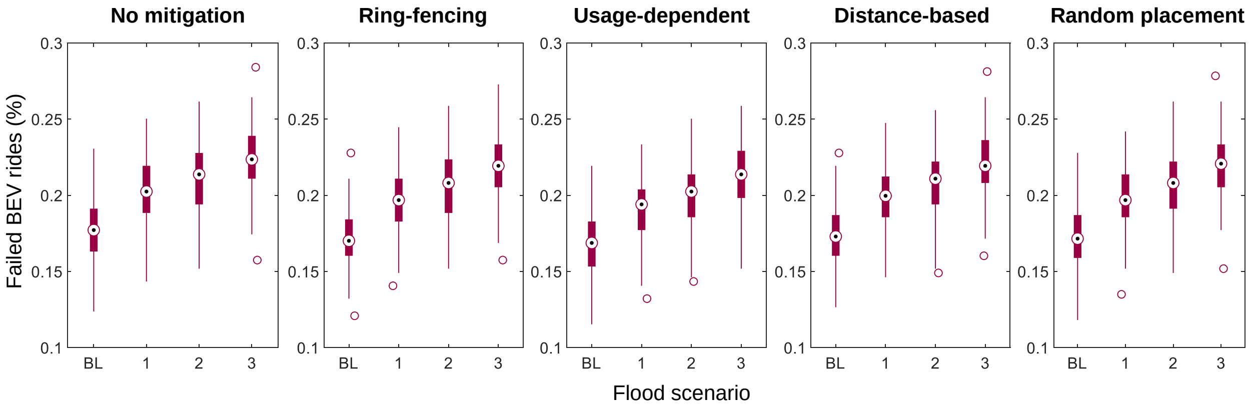

Supplementary Figure 15. Failure rate of BEV rides for different mitigation strategies. The central mark, bottom, and top edges of each box plot represent the median, 25th, and 75th percentiles, respectively. Outliers, if any, are marked as individual circles. [BL: Baseline scenario with no flooding]

3.2 Variation of Fig. 4 for different flood scenarios and number of chargers added

Fig. 4 in the main article presented the results for the four flood mitigation strategies while considering flood scenario-2 and that 5% additional chargers were added. This section presents the same results, but (i) for flood scenarios-1 and -3 and 5% additional chargers, and (ii) when the number of newly added chargers is changed to 2.5% and 10% for flood scenario-2.

3.2.1 Different flood scenarios

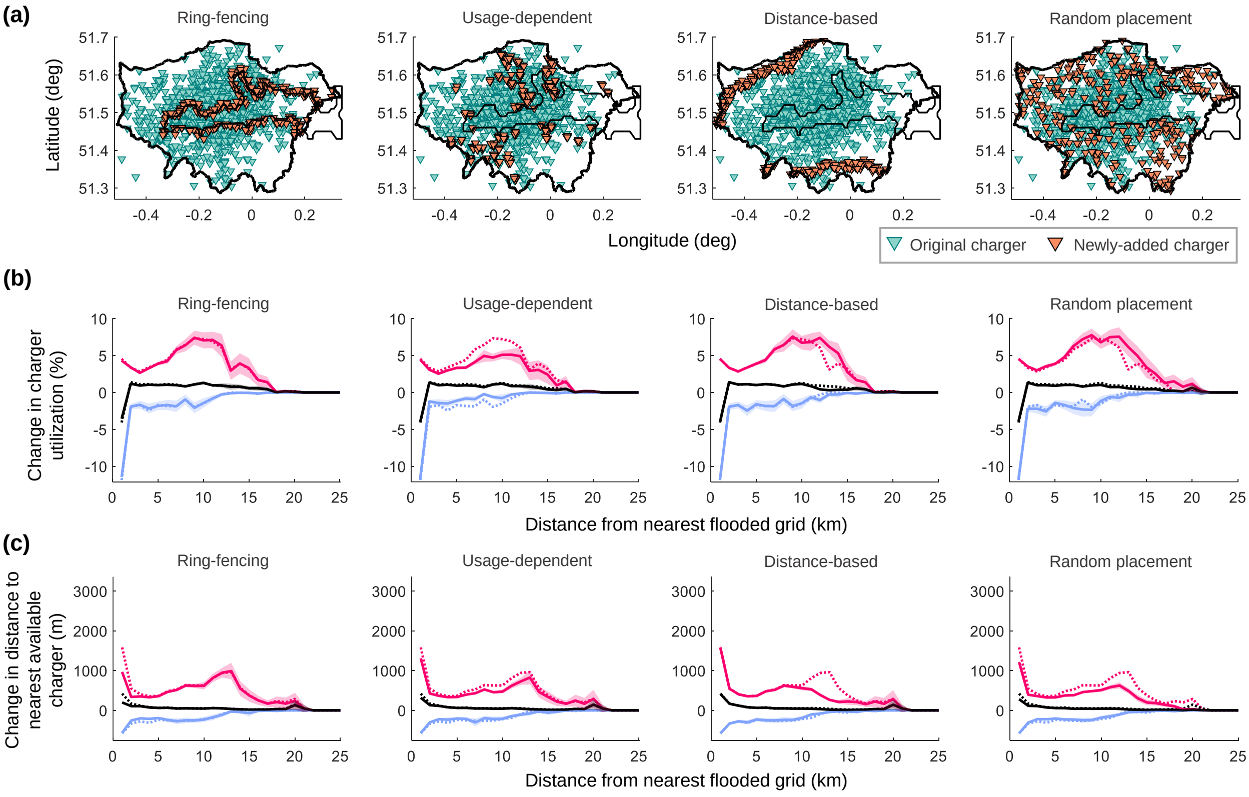

Supplementary Figure 16. The same as Fig. 4 in the main article, but for flood scenario-1.

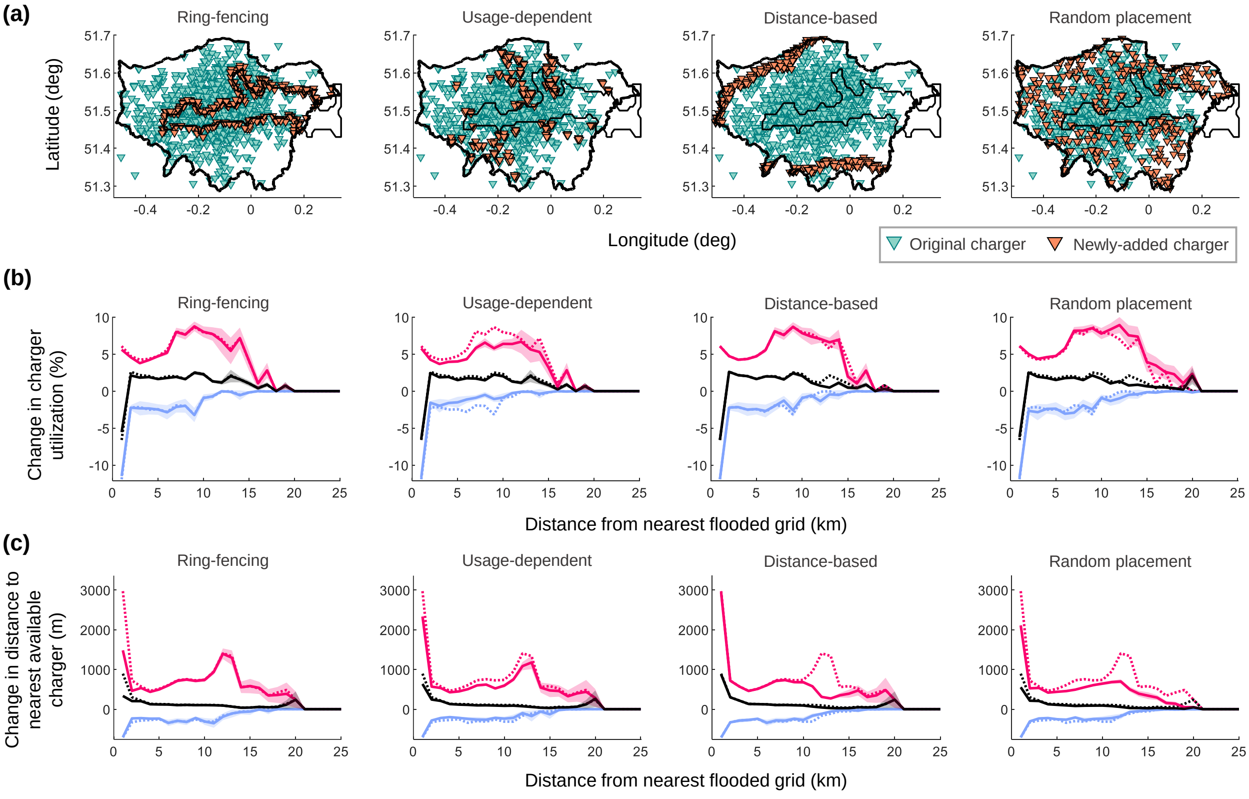

Supplementary Figure 17. The same as Fig. 4 in the main article, but for flood scenario-3.

3.2.2 Different number of chargers added

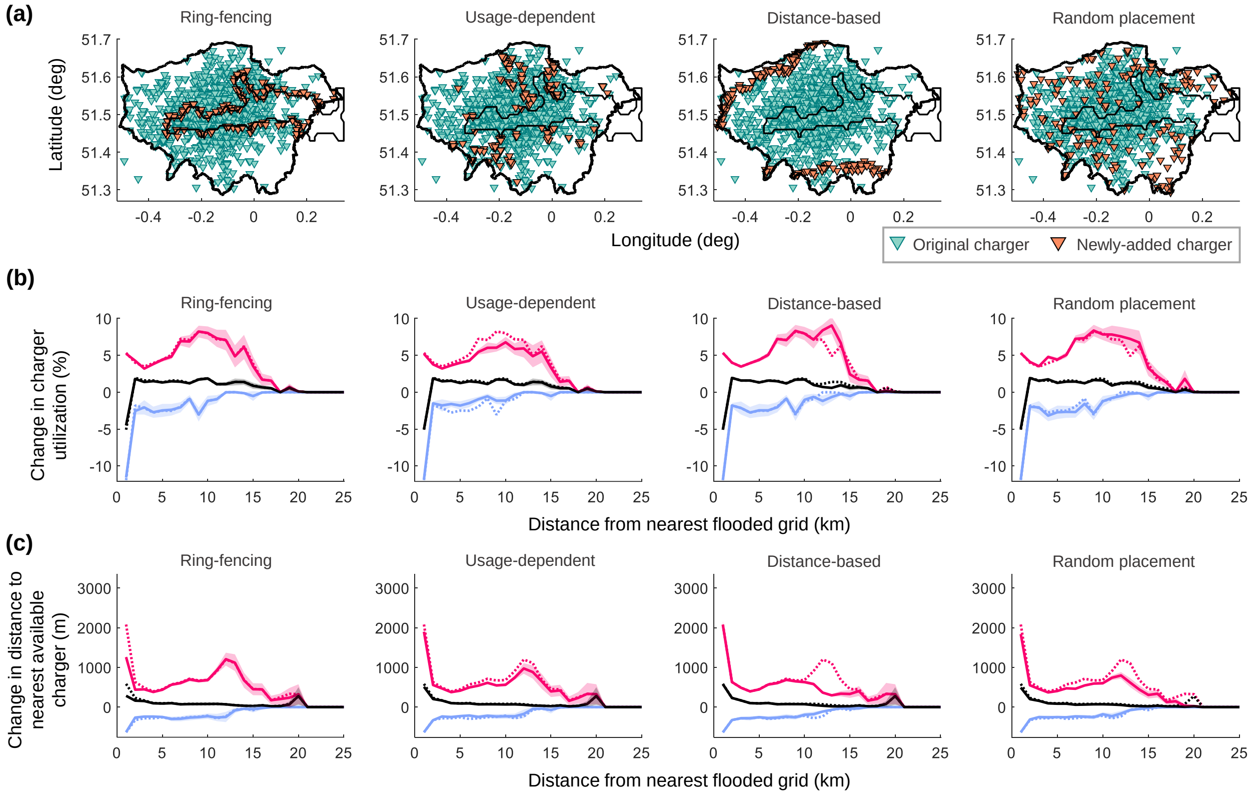

Supplementary Figure 18. The same as Fig. 4 in the main article, but for 2.5% added chargers.

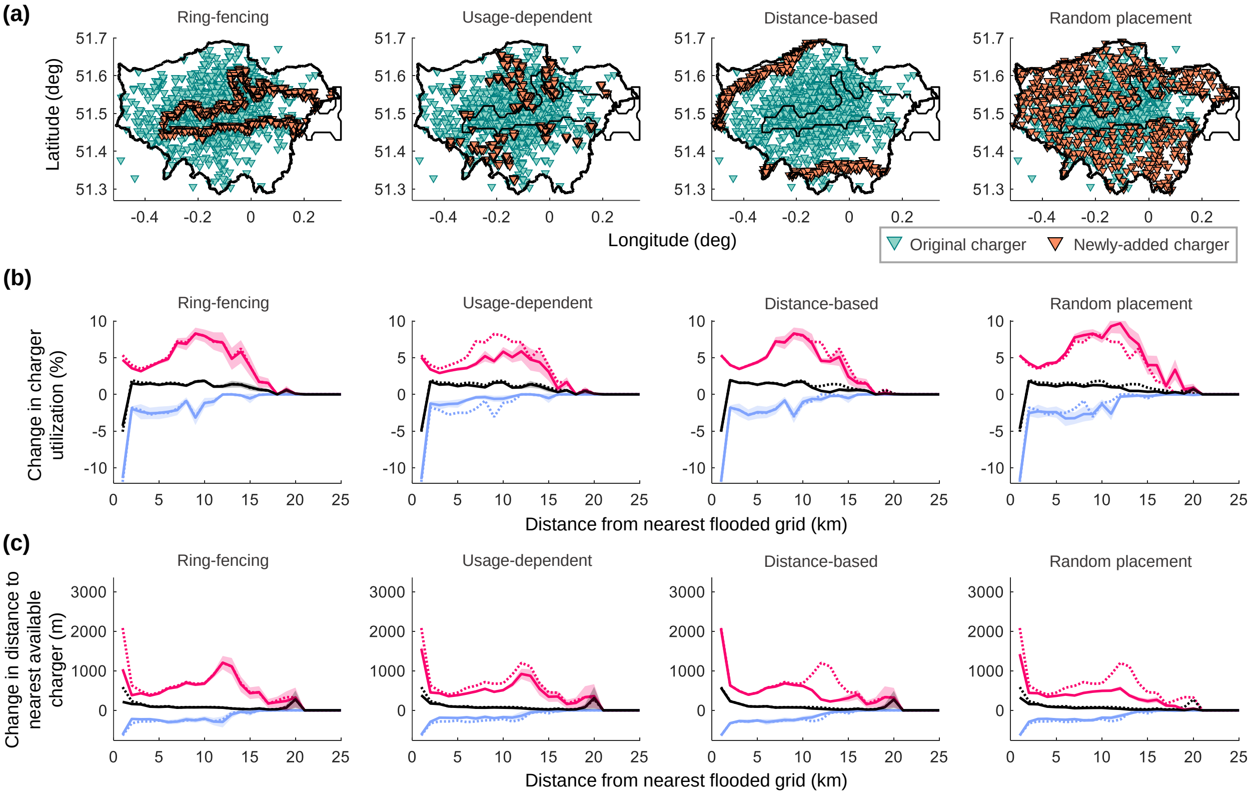

Supplementary Figure 19. The same as Fig. 4 in the main article, but for 10% added chargers.

4 Supplementary note 4: Locations of public EV chargers in Greater London

We obtained the locations of the public EV chargers in Greater London from the website maintained by the Office of the Mayor of London [1], which consists of all the locations of slow (defined as < 43 kW) and rapid chargers (defined as ≥ 43 kW). Overall, there were 5,925 chargers located across the region at the time of our accessing, see Supplementary Figure 20. This dataset is available for download at ref. [2].

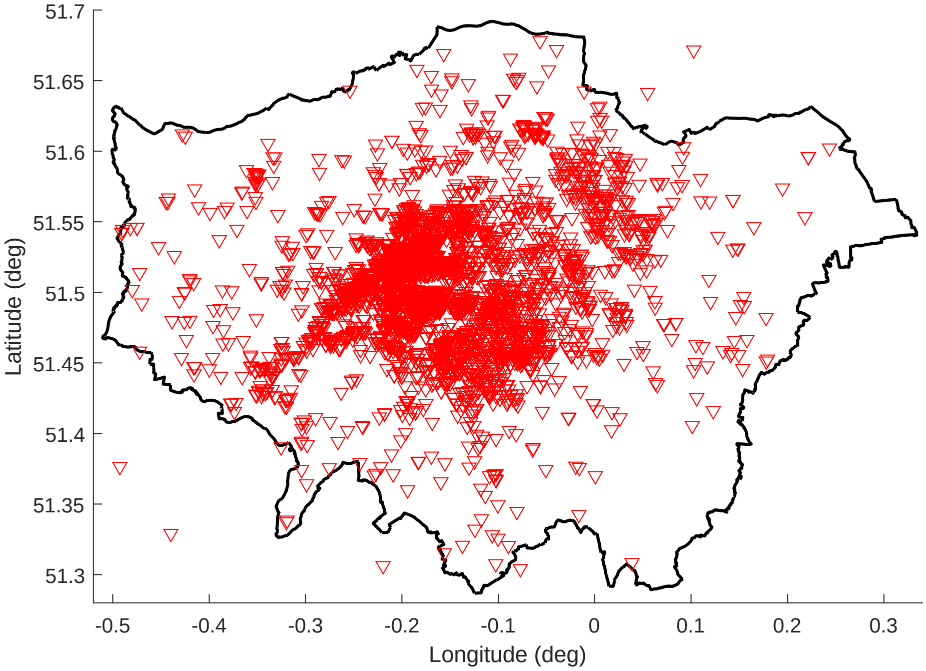

Supplementary Figure 20. Location of the public chargers used in this study.

5 Supplementary note 5: Simulation methodology

Supplementary Figure 21 shows a flowchart of the simulation methodology adopted in our study. Details of each step are presented in the Methods Section of the main article.

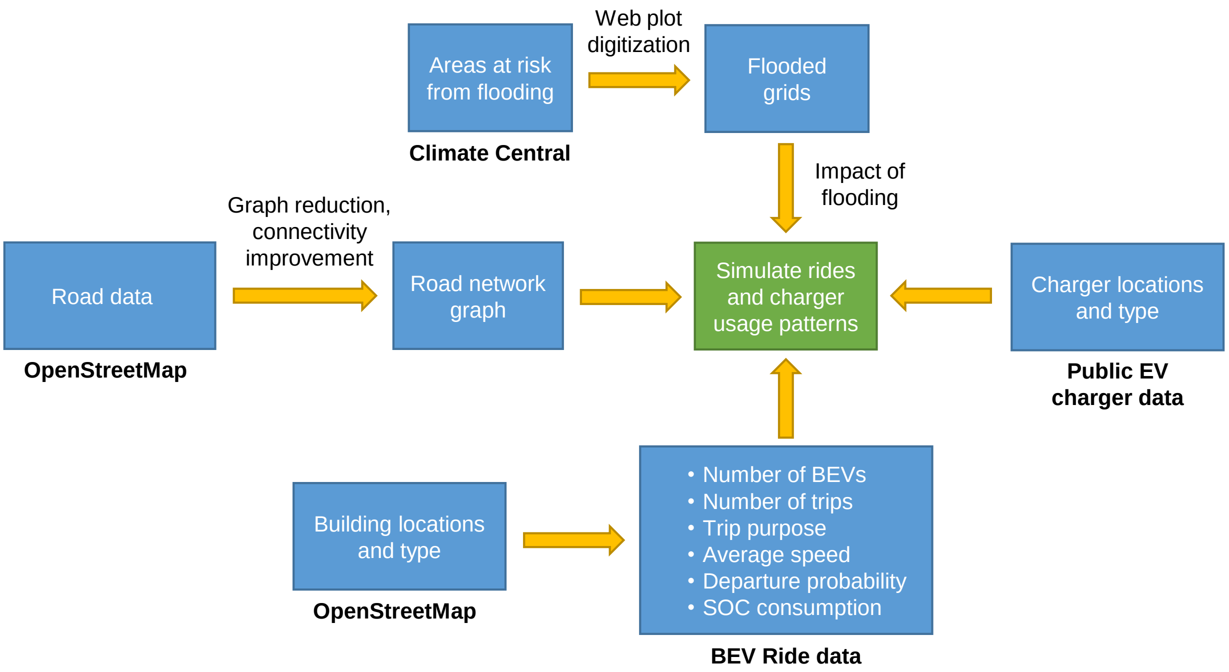

Supplementary Figure 21. Flowchart of the simulation methodology.

6 Supplementary note 6: Simulating BEV rides

This section presents additional data used to simulate the rides for the BEVs in our study. The data is publicly available at ref. [3]. Supplementary Table 1 presents the probabilities for different number of trips taken by each BEV throughout the day. Given the number of trips, the intermediate destinations of the BEVs are selected as per the probabilities in Supplementary Table 2. Finally, Supplementary Table 3 presents the ranges of the average speed at various times of the day.

Supplementary Table 1. Probability distribution of number of trips for each BEV during the day

Number of trips	Probability
2	0.720
3	0.170
4	0.080
5	0.020
6	0.005
7	0.005

Supplementary Table 2. Selection of destination type for tours

<table>
<tr><th>Type of tour</th><th>Probability</th><th>Type of destination building</th></tr>
<tr><td rowspan="2">Single-purpose tour (trips=2)</td><td>23/82</td><td>Work (Sole destination)</td></tr>
<tr><td>59/82</td><td>Commercial (Sole destination)</td></tr>
<tr><td rowspan="2">Multi-purpose tour (trips≥3)</td><td>8/18</td><td>1 Work, 1 Commercial, and the rest, random</td></tr>
<tr><td>10/18</td><td>all Commercial</td></tr>
</table>

Supplementary Table 3. Speed range for different hours of the day

Time interval	Classification	Average speed range (km/h)
4 AM- 7 AM	Intermediate	[13.2, 34.5]
7 AM- 10 AM	AM	[14.5, 30.8]
10 AM- 5 PM	Intermediate	[13.2, 34.5]
5 PM- 11 PM	PM	[13.7, 29.7]
11 PM- 4 AM	Intermediate	[13.2, 34.5]

The probability distributions of departures throughout the day for work and commercial purposes is shown in Supplementary Figure 22.

6.1 BEV charge and discharge rate

The ranges of the different BEV models considered here are as follows, as per the EPA Federal Test Procedure: Nissan Leaf (40 kWh battery): 243 km [4], Nissan Leaf Plus (62 kWh battery): 364 km [4], and Tesla Model S (100 kWh battery): 603.5 km [5]. For each model, it is assumed that the full range of battery state-of-charge (SOC) from 100% (full charge) to 20% (minimum allowed value) yields the aforementioned range. During a trip, the BEV is assumed to linearly discharge at the rate:

$$\mathcal{D} = \frac{1 - 0.2}{d_{max}} = \frac{0.8}{d_{max}}, \tag{1}$$

where d_{max} is the range of the BEV.

Along similar lines, the charging rate is calculated as:

$$\mathcal{C} = \frac{0.8}{E_{max}} \eta_c P_c, \tag{2}$$

where E_{max} is the battery capacity, η_c the charging efficiency (taken here as 0.89 for all chargers), and P_c the charger power capacity.

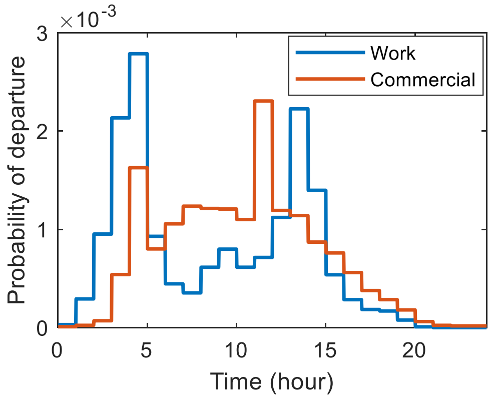

Supplementary Figure 22. Departure probabilities throughout the day for different trip purposes. Source: [3].

6.2 Ride distances

The results of our ride simulations are presented in Supplementary Figure 23, showing the distribution of ride distances over 100 random simulations. In particular, this figure presents the aerial distance between the different source-destination pairs for all the rides in each simulation, calculated using the Haversine method. Overall, the average Haversine distance of a trip in our simulations is 15.8 km, which corresponds well to the value of 13.9 km obtained in prior surveys [3, 6] in Greater London.

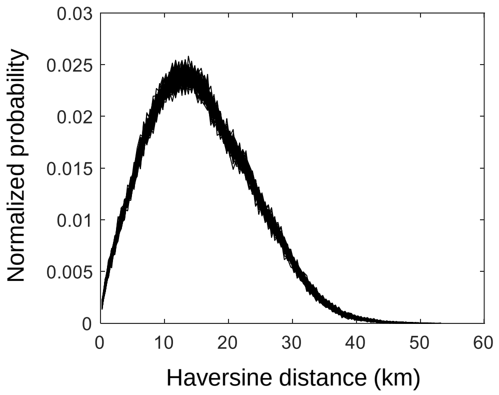

Supplementary Figure 23. Distribution of the Haversine distance of trips for 100 simulations, with the number of BEVs to public chargers equal to 6.

7 Supplementary note 7: Areas at risk from flooding in Greater London

This section presents a comparison of the regions at risk from flooding in Greater London obtained from: (i) the coastal risk screening tool by Climate Central which models sea level rise and coastal flooding and is used in our simulations; and (ii) the estimates of flood risk from the UK Environment Agency [7], which includes the effect of existing flood defenses and flooding from river and surface water. The estimates from these two sources are presented in Supplementary Figure 24, which shows largely overlapping areas. The former area corresponds to a 10% risk of flooding per year, and the latter includes regions with a flooding risk of >0.1% and >3.3% per year.

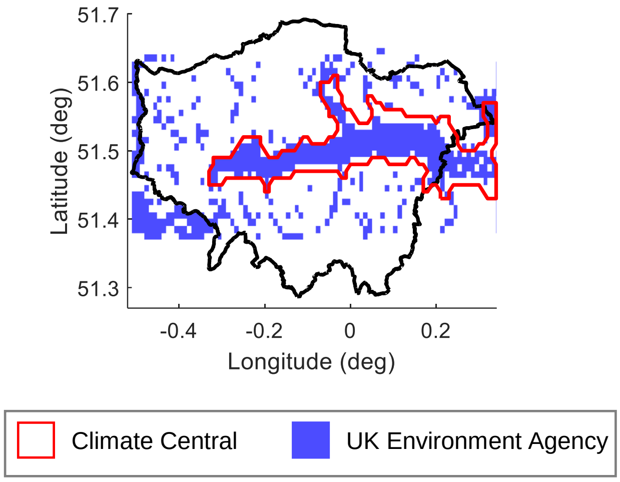

Supplementary Figure 24. Comparison of at-risk areas for flooding in Greater London.

Furthermore, we compare the number of chargers that are affected by flooding over 100 random simulations for the three flood scenarios and the two datasets, see Supplementary Table 4. Here again, we find only minor differences.

Supplementary Table 4. Percentage of chargers affected by flooding in the three scenarios [BL: Baseline scenario with no flooding, FS: flood scenario]

	Climate Central				UK Environment Agency			
	BL	FS-1	FS-2	FS-3	BL	FS-1	FS-2	FS-3
Minimum	0	13.0	17.9	27.0	0	12.8	19.0	27.5
Median	0	17.0	24.6	32.0	0	16.7	23.8	30.3
Maximum	0	23.9	28.8	34.3	0	20.7	27.4	32.7

References

[1] London electric vehicle charging points. https://www.arcgis.com/home/webmap/viewer.html?url=https%3A%2F%2Fgis.london.gov.uk%2Farcgis%2Frest%2Fservices%2Fapps%2Fev_charge_points%2FMapServer&source=sd. Accessed on: 21/10/2021.

[2] Supporting data. https://www.penglaboratory.com/ev-charger-flooding-data, 2021.

[3] Transport for London. Travel in London, Supplementary Report: London Travel Demand Survey (LTDS). https://www.clocs.org.uk/wp-content/uploads/2014/05/london-travel-demand-survey-2011.pdf, 2011. Accessed on: 28/5/2021.

[4] Nissan Leaf. https://en.wikipedia.org/wiki/Nissan_Leaf. Accessed on: 25/10/2021.

[5] Tesla Model S. https://en.wikipedia.org/wiki/Tesla_Model_S. Accessed on: 25/10/2021.

[6] Transport for London. Roads Task Force – technical note 12: How many cars are there in London and who owns them? http://content.tfl.gov.uk/technical-note-12-how-many-cars-are-there-in-london.pdf, 2011. Accessed on: 28/5/2021.

[7] UK Environment Agency. Risk of flooding from multiple sources: Risk band. https://data.gov.uk/dataset/0afc0a17-cb2c-4221-bcb8-947e61ac30f0/risk-of-flooding-from-multiple-sources-risk-band, 2021. Accessed on: 10/02/2022.
